# Charting the landscape of the environmental exposome

**DOI:** 10.1002/imt2.50

**Published:** 2022-09-02

**Authors:** Xin Wei, Zinuo Huang, Liuyiqi Jiang, Yueer Li, Xinyue Zhang, Yuxin Leng, Chao Jiang

**Affiliations:** ^1^ Zhejiang Provincial Key Laboratory of Cancer Molecular Cell Biology, Life Sciences Institute Zhejiang University Hangzhou Zhejiang China; ^2^ Department of Genetics Stanford University Stanford California USA; ^3^ Department of Intensive Care Unit Peking University Third Hospital Beijing China; ^4^ Zhejiang Provincial Key Laboratory of Pancreatic Disease, First Affiliated Hospital Zhejiang University School of Medicine Hangzhou Zhejiang China

**Keywords:** exposome, environments, chemicals, microbes

## Abstract

The exposome depicts the total exposures in the lifetime of an organism. Human exposome comprises exposures from environmental and humanistic sources. Biological, chemical, and physical environmental exposures pose potential health threats, especially to susceptible populations. Although still in its nascent stage, we are beginning to recognize the vast and dynamic nature of the exposome. In this review, we systematically summarize the biological and chemical environmental exposomes in three broad environmental matrices—air, soil, and water; each contains several distinct subcategories, along with a brief introduction to the physical exposome. Disease‐related environmental exposures are highlighted, and humans are also a major source of disease‐related biological exposures. We further discuss the interactions between biological, chemical, and physical exposomes. Finally, we propose a list of outstanding challenges under the exposome research framework that need to be addressed to move the field forward. Taken together, we present a detailed landscape of environmental exposome to prime researchers to join this exciting new field.

## Highlights

- The biological and chemical exposures in air, soil, and water were summarized and united under the exposome framework.
- Biological, chemical, and physical exposures are dynamically interweaved.
- A list of outstanding challenges was proposed to be tackled to push the field forward.

## Graphical abstract

We describe the biological and chemical components of the environmental exposomes in three major environmental matrices that are highly relevant to human and social‐economical health—air, soil, and water. We discuss how different exposome components can interact with each other. Finally, we propose a list of outstanding challenges to be tackled to push the field forward.

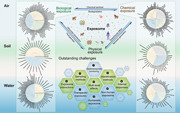

## INTRODUCTION

**Figure 1:**
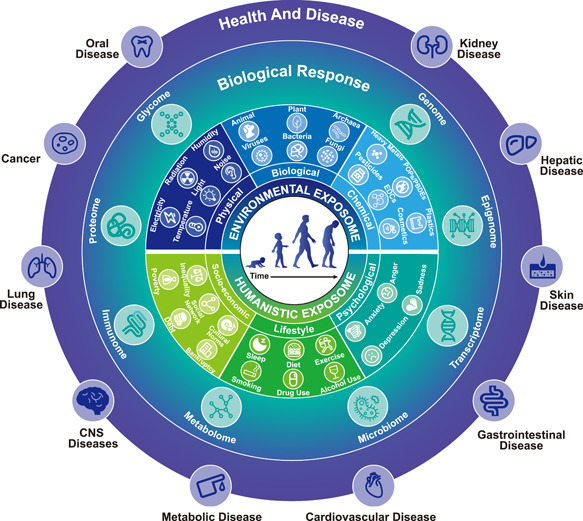
Exposome and its impact on health (using the human as an example). The environmental exposome includes physical, biological, and chemical exposomes, including components such as noise, temperature, bacteria, viruses, pesticides, and heavy metals. The humanistic exposome includes socioeconomic, lifestyle, and psychological exposomes, including components, such as inequality, poverty, diet, smoking, anxiety, and depression. The exposome can impact the biology of all organisms (using the human as an example), from gene expression to metabolic changes, leading to adverse or beneficial effects on health. CNS, central nervous system; EDCs, endocrine‐disrupting chemicals; PBDEs, polybrominated diphenyl ethers; POPs, persistent organic pollutants.

The totality of exposures plays a pivotal role in the dynamic balance between health and disease in humans and all organisms [1, 2]. Proposed by the cancer epidemiologist Christopher Wild in 2005 [3], the original concept of exposome is to encompass all environmental exposures during the entire life of an individual, from womb to tomb. In 2012, the scope of exposome had been further expanded to comprise three major domains: internal, specific external, and general external [4], which integrates the internal environment of the body (biological response), the specific external agents to which one is exposed (environmental exposome), and the social, cultural, and ecological contexts in which the person lives their life (humanistic exposome), as shown in Figure 1. As a critical counterpart to the genome, the proposition of exposome is intended to draw attention to identifying and evaluating nongenetic factors and their impact on health.

Ingestion, skin‐contact, and inhalation are three major exposure pathways. Specifically, (1) environmental exposures can enter our body along the digestive tract through food and drink and can have long‐term effects on human health. Natural food and drink (composed mainly of plants, animals, fungi, and other microbes) are also exposed to environmental exposures, which can indirectly affect human health. For example, heavy metal contaminations in soil–food crop systems adversely impact food security and human health, disturbing human metabolomics, and contributing to morbidity and even mortality [5, 6]. (2) Common types of environmental exposure via skin‐contact include solar exposure and air pollution. Photoaging, photocarcinogenesis, and pigmentation are recognized consequences of long‐term skin exposure to solar radiation. Exposure to traffic‐related air pollution can cause skin aging. Particulate matter (PM) and nitrogen dioxide (NO_2_) can cause skin pigmentation/moles, while ozone (O_3_) can cause wrinkles and affect atopic eczema [7]. (3) Air pollutants are ubiquitous (e.g., volatile organic compounds [VOCs]) either indoors or outdoors [8, 9], which poses a great danger for populations who are chronically exposed to a high concentration of VOCs at work [10]. Exposure to VOCs may cause irreversible health effects [11]. Some VOCs, such as benzene, 1,3‐butadiene, and vinyl chloride, are classified as Group 1 human carcinogens by the International Agency for Research on Cancer [12]. In addition, inhalation of bioaerosols carrying fungal particles (e.g., *Aspergillus*) can cause various symptoms, such as asthma, respiratory infections, allergic pneumonia, allergic rhinitis, and bronchitis [13].

Exposure–health relationships have already been carefully investigated for decades, especially in the fields of public health [14], environmental toxicology [15], medical science [16, 17], environmental chemistry [18], and psychology [19]. Earlier efforts usually did not take omics approaches toward characterizing diverse environmental exposures or the physiological consequences of the exposures and were often centered around humans. The next‐generation sequencing (NGS) and mass spectrometry (MS) technologies have boosted the exposome research into the omics era. Increasing studies are revealing the impacts of the exposome on transcriptomics, metabolomics, proteomics, immunomics, epigenetics, glycomics, genomics, etc. [4, 20, 21, 22, 23] (Figure 1), which provides in‐depth and unique insights into the relationships between the exposome and diseases [24]. Several experts in the exposome field have systematically reviewed the relationships between environmental exposome and human diseases, such as asthma [25], cardiovascular diseases [26], pregnancy [27], cancer [28, 29, 30], gastrointestinal disease [31], skin disease [7], kidney disease [32], metabolic disease [33, 34], and other health conditions [35, 36]. Besides environmental exposures, humanistic exposome comprising lifestyle and socioeconomic factors also play important roles in defining and shaping one's health (Figure 1) [37, 38]. Scientists are still trying to define what the lifestyle and socioeconomic exposome comprise and how to quantify them. Some parts of the humanistic exposome can be tracked by wearable or portable devices, which is emerging as an exciting research field [39, 40, 41, 42, 43].

The addition of exposome to the equation of disease–health dynamics sparks several new fields for future research. However, precisely what, when, where, and how the exposures were encountered is less understood. At the forefront, there is an urgent need for free and easily accessible databases for chemical and biological exposome quantification, reference‐dependent or ‐independent identification methods of unknown exposures and their properties (to answer the WHAT); a systematic network of exposome monitoring devices, and the development of efficient wearable devices for individual use (to answer the WHEN and WHERE); rigorous experimental design and advanced statistical methods to analyze the often spatiotemporally variable exposome data, and established cell‐line and experimental animal systems to investigate the impact of the exposome in different types of acute and chronic diseases at the mechanistic level (to answer the HOW). Conceptually, the impact of environmental exposures even goes beyond organisms and extends to abiotic objects [2].

Given the enormous scope of the exposome, the goal of this review is not to discuss all aspects of exposome research, some of which have been extensively reviewed recently [2, 37, 44, 45]. Instead, we aim to take this opportunity to summarize and unite the main types of environmental exposures in major environmental matrices, as revealed by decades of research, under the exposome framework and discuss how the exposures are interconnected. Specifically, we focus on the biological and chemical components of the environmental exposomes in air, soil, and water, which are highly relevant to human and social‐economical health. We discuss how different exposome components can interact with each other. Finally, we propose a list of outstanding challenges to be tackled to push the field forward.

## AN ATLAS OF THE ENVIRONMENTAL BIOLOGICAL EXPOSOME

**Figure 2:**
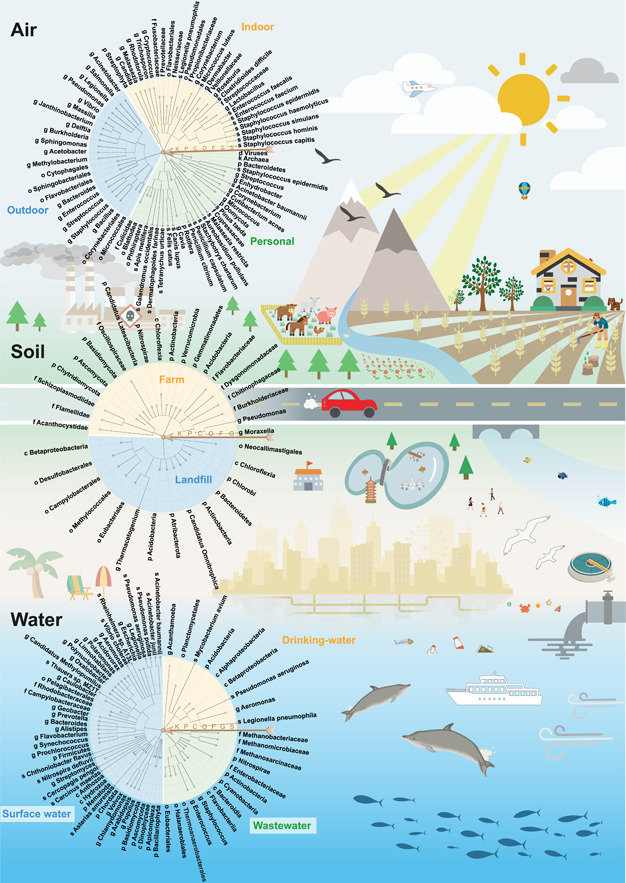
Different biological exposures in air, soil, and water matrices. Summarized biological exposures in different environmental matrices are represented in the form of assembled taxonomic trees (circles on the left). Each circle is assembled by two or three taxonomic trees based on distinct subenvironments (represented by different colors within a circle). Trees are constructed by a homemade tool using a summarized taxa identifier as input based on the NCBI taxonomy database (https://ftp.ncbi.nih.gov/pub/taxonomy/) and visualized using the online tool iTOL (https://itol.embl.de/) [63]. Arrows with letters denote the corresponding taxonomic levels. Potential sources of biological exposures are also illustrated. C, class; F, family; G, genus; K, kingdom; O, order; P, phylum; S, species.

Biological exposures in the environmental exposome have been historically studied in the contexts of different fields, including ecology [46, 47], infectious diseases [48, 49, 50], public health [51, 52], and more recently, microbiome [53, 54, 55]. More research has focused on the harmful impact of biological exposures, but biological exposures are not necessarily adverse [56]. Some have been demonstrated to be beneficial, especially considering the human microbiome part of the exposome [57]. Biological exposures were not always studied from a human‐centric perspective. For example, ecologists may be interested in profiling the diversity of microbial species in the air [58], water [59], and land [60]. Still, they are not necessarily concerned about how these diverse microbial species could impact the health of humans or other organisms. On the other hand, in the medical field of infectious diseases, great efforts and resources have been dedicated to studying how a few species of pathogens can invade human organs or cells at the molecular level, without much attention to other organisms that cohabit in the same environment [61, 62]. We take a more systematic view by considering the environmental biological exposures to be highly diverse and dynamic, comprising at least thousands of species across all super‐kingdoms/kingdoms of organisms: Bacteria, Fungi, Viridiplantae, Metazoa, Viruses, and Archaea. Below we summarize the major types of biological exposures in air, water, and soil environmental matrices (Figure 2 and Supporting Information Table S1).

### Air

Millions of bioaerosols surround us daily, which can negatively and positively impact our health [64]. The broader category of bioaerosols includes, for example, whole microorganisms, pollen, protozoan, tiny invertebrates, skin flakes, or traces of insects. A more comprehensive spectrum of airborne biological components has been characterized recently [39]. Advances in sampling techniques and NGS led to characterizations of the airborne exposures of various environments, including indoor air [65], outdoor air [66], and personal air exposures [39, 67].

#### Indoor airborne biological exposome

Humans spend around 90% of their time indoors, the exposome of the built environment is of particular concern [68]. Indoor airborne exposures are complex and have eight primary sources: humans, outdoor environments, plumbing systems, plants, molds, pets, heating, ventilation, and air‐conditioning systems; and dust resuspension [64]. The factors influencing indoor air exposome are mainly human occupancy and building‐related factors, including ventilation, airflow direction, temperature, and humidity [69, 70, 71]. Below we mainly focus on indoor biological exposures originating from humans, outdoor environments, and plumbing systems.

As one of the most contributed sources of indoor air exposure, there are about 10^12^ microbes on our epidermis and 10^14^ microbes in the digestion tract [64]. Microbes belonging to *Corynebacteriaceae*, *Fusobacteriaceae*, *Neisseriaceae*, *Prevotellaceae*, *Propionibacteriaceae*, *Staphylococcaceae*, and *Veillonellaceae* were abundant in the upper respiratory tract of a healthy human. Most of them have also been detected in indoor air [72]. In the air of an office building, human‐associated *Micrococcus*, *Staphylococcus*, and *Streptococcaceae* were the most representative flora [73]. These examples indicate that human occupancy contributes to the characteristics of the indoor airborne exposome. Furthermore, researchers suggested that there might be differences in the microbiome composition due to male or female occupancy. *Corynebacterium*, *Dermabacter*, and *Roseburia* had higher relative abundances in homes occupied by males. Homes occupied by females were dominated by *Lactobacillus*, which is abundant in the female genital tract [74]. Besides bacteria, some skin‐associated fungal groups can be released into the air upon shedding. Yamamoto et al. [75] found that floor dust in classrooms was enriched in skin‐associated yeasts, such as the genera *Rhodotorula*, *Candida, Cryptococcus, Malassezia*, and *Trichosporon*. Interactions among humans in closed environments can further amplify the human impact on the air exposome. For example, a super spreading event of COVID‐19 occurred within nine days in 3 of 6 cubicles at a general ward with no air exhaust built within the cubicles. This event involved nine healthcare workers (HCWs) and 12 patients and was potentially attributed to the airborne transmission of SARS‐CoV‐2 lineage B.1.36.27 among patients and HCWs [76].

Ventilation can influence the indoor exposome by affecting air circulation indoors and outdoors [77]. In a well‐ventilated built environment with moderate occupancy, outdoor air may have a more significant impact on microbial composition than human habitation. In a room with natural ventilation, >88% of the microbial taxa is present in indoor and outdoor environments, including environmental fungi *Mycosphaerella* and *Cladosporium* [78]. Adams et al. [79] found that some outdoor‐associated taxa had high abundances in a mechanically ventilated, office‐like building, such as Burkholderiales, Pseudomonadales, Flavobacteriales, and Streptophyta.

The plumbing systems of a building can generate aerosols via showerheads, toilets, faucets, and kitchen sinks [68]. The aerosols from showerheads can contribute to the increased density of opportunistic pathogens inhabiting water systems; it is estimated that *Legionella* is greater than 10^2^ CFU/m^3^ in shower air [80]. Aerosols with microbes can also be generated after flushing the toilet [81]. Fecal pathogens (e.g., *Clostridium difficile*) can be identified in aerosols collected from 25 cm above the toilet after frequently flushing and even 90 min after the most recent flush [82].

Taken together, the composition of indoor airborne biological exposures is mainly derived from a mixture between human‐related activities and outdoor air, given that ventilation is sufficient.

#### Outdoor airborne biological exposome

Diverse microbial entities (e.g., bacteria, fungi, archaea, protozoa, and viruses) and organismal fragments or excretions have been identified within the bioaerosols [83]. Both natural processes (e.g., pollination, wildfires, volcanic eruption, desert dust, and sea spray) and human activities (agriculture and industry) can be the sources of bioaerosols [84].

Generally speaking, the dominant bacterial phylum in outdoor air is Proteobacteria, composed of representative orders, including Pseudomonadales, Burkholderiales, Rhizobiales, Rhodospirillales, and Sphingomonadales. These orders are further decoded at the genus level, including *Pseudomonas* and *Acinetobacter*; *Massilia*, *Delftia*, and *Janthinobacterium*; *Methylobacterium*; *Acetobacter*; and *Sphingomonas*, respectively [85]. Besides Proteobacteria, other phyla were frequently found in outdoor air, including Firmicutes, Actinobacteria, and Bacteroidetes. These phyla were dominated by orders Bacillales and Lactobacillales; Corynebacteriales and Micrococcales; and Sphingobacteriales, respectively.

Seasonal and spatial/geographic factors drive the dynamic changes in outdoor airborne exposome [86]. These factors include temperature, humidity, wind speed, population density, and altitude. Studies have shown that bacterial diversity in outdoor air is highest in summer [87, 88]. The diversity and composition of airborne bacteria vary according to the location of the sampling area. Urbanization can lead to the homogenization of outdoor air microbial communities, with less geographic variability in urban environments than in rural areas [86, 89, 90]. In coastal areas, the order Flavobacteriales of the phylum Bacteroidetes was more representative, while in inland regions, the orders Bacteroidales, Cytophagales, and Sphingobacteriales were more representative [83].

A few pathogenic bacteria were found in outdoor bioaerosols. Most known bacterial pathogens are *Acinetobacter*, *Bacillus*, *Bacteroides*, *Burkholderia*, *Enterococcus*, *Pseudomonas*, *Streptococcus*, *Staphylococcus*, and *Vibrio* [88]. Air bacteria that pose a severe threat to human health, such as *Bacillus anthracis*, *Legionella*, and *Salmonella*, have been founded in composting facilities, dust storms, and urban areas [83, 91, 92]. Additionally, airborne bacterial pathogens around the hospitals and sewage treatment plants are more diverse and abundant than in areas farther away from these locations [93]. Interestingly, irrigation with recycled water and fertilizing with sewage sludge can increase the abundance of bacterial pathogens in the city and adjacent suburbs [88].

In summary, the outdoor airborne biological exposures comprise microbes from natural processes and human activities. Both meteorological (e.g., seasonal factors and spatial/geographic location) and anthropogenic factors (e.g., industrialization and urbanization) drive the dynamics of outdoor airborne biological exposures.

#### Personal dynamic airborne biological exposome

Historically, researchers have been monitoring air particulates and toxins using immovable or cumbersome sampling stations in distinct regions to assess how these exposures affect the population's heath [94, 95]. However, personal air exposome is highly dynamic and heavily influenced by personal lifestyle in addition to the aforementioned general meteorological and anthropogenic factors. Scientists proposed the personal exposome monitoring scheme to unravel what humans are exposed to in their daily lives. A recent study has revealed the exact composition of the airborne exposome at the individual level using a wearable collection device [39].

The longitudinal personal airborne exposome study by Jiang et al. [39] identified 2560 species, 1265 genera, and 44 phyla from the samples, consisting of taxa belonging to Bacteria, Fungi, Metazoan, Viridiplantae, Viruses, and Archaea. Seasonal patterns can be found among these taxa. For example, the green leaf plant's phylum, Streptophyta, was most abundant in spring and summer. The phylum of yeasts and most molds, Ascomycota, increased in summer and fall, while Basidiomycota (which includes all mushrooms) was the dominant fungal phylum during winter and spring. Significant changes were found in ascomycetes when comparing the campus samples with noncampus samples, suggesting that they were most influenced by the location/lifestyle. Five human‐related bacterial genera (*Corynebacterium*, *Enhydrobacter*, *Staphylococcus*, *Streptococcus*, and *Rothia*) were representative in noncampus samples. These findings demonstrate that personal exposome varies significantly spatiotemporally, and more work needs to be done to have a better general idea of exposome at the individual level.

### Soil

Soil is home to a highly diverse and complex biological community, including bacteria, archaea, fungi, protozoa, viruses, and more complex organisms (plants, insects, nematodes, etc.) [96]. Wind, rain, and daily outdoor/indoor activities constantly expose humans to microbes living in the soil, which is especially obvious for playful toddlers that rely more on the upper body to move around. In addition, soil microbes can be easily delivered to our dining tables through dairy products, meat, crops, and vegetables if not carefully handled and sanitized. Recent findings indicate that exposure to various soil‐derived microbes may be beneficial to the development of the immune system of infants over the long haul [97, 98]. The health implication of soil microbe exposure is further compounded by the fact that a significant portion of the world population still uses manure as a source of fertilizer, therefore connecting the gut microbiomes of animals and humans directly to the soil‐derived exposome.

#### Farm and rhizosphere

Microorganisms are invisible engines of soil fertility [99]. For example, bacteria and fungi can mineralize nutrients and supply them to plants. Microbes secrete sticky polysaccharides that hold soil particles together and prevent erosion. They also work together to regulate the hormonal balance of plants, help plants cope with abiotic stressors, and protect them from a range of pests, parasites, and pathogens [100, 101]. Agricultural soil, especially soil in the rhizosphere, is directly adjacent to and affected by roots and has high microbial biomass and species diversity. It is estimated that 1 g rhizosphere soil contains 10^8^–10^11^ culturable cells and approximately 10^4^ microbial species [100].

The influence of regional and spatial distribution on the composition of soil exposures is of primary importance in soil science. A study analyzed soil samples from 200 vineyards on four continents, representing microbial biogeographical patterns on a global scale [102]. The evaluation of fungal taxa showed that *Solicoccozyma* was the dominant genus in the vineyards of Argentina, Chile, Croatia, South Africa, and Italy, with relative abundances ranging from 13.4% to 39.3%. In Portuguese and South African vineyards, *Fusarium* and *Cladosporium* were the most dominant genera with a relative abundance of up to 10%.

Seasonal changes in temperature and humidity greatly influence the bacterial community structure and abundance in soil. A study showed that the bacterial diversity of abandoned cropland was higher in the growing period (March–September) than in the resting period (October–February) [103]. The relative abundance of Bacteroidetes and Verrucomicrobia increased during the growing period, while Actinobacteria and Chloroflexi had an increased abundance during the resting period. These findings reveal the seasonal dynamics of the soil microbial community [103].

Agricultural activities have a significant influence on soil exposome. Among them, tillage, irrigation, fertilizers, and changes in crop species can affect physical, chemical, and biological processes in the soil [104]. As soil microbes are essential to the continued productivity of sustainably managed agroecosystems, some agriculture‐related soil microbiome studies have focused on investigating the effects of fertilization and improvement strategies on farm microbiome [105, 106, 107]. Below, three studies on mesocosm experiment, dairy farm, and coffee plantation are summarized as examples to reveal the representative farm taxa groups.

A 1‐year long mesocosm experiment was performed by Cesarano et al. [105] to investigate soil microbial communities' compositions under different organic amendments strategies. Thirty bacterial phyla were detected in all samples, including Acidobacteria, Actinobacteria, Bacteroidetes, Chloroflexi, Firmicutes, Gemmatimonadetes, and Proteobacteria. The growth of Acidobacteria and Gemmatimonadetes bacteria was promoted by adding organic amendments. On the contrary, Actinobacteria and Proteobacteria were more abundant in the soil treated with synthetic fertilizer. Compared with the use of synthetic fertilizers, the application of organic materials can improve the diversity and functionality of the microbial community.

Manure has been widely used as fertilizer because of its nutrient‐rich and stable organic carbon composition. To evaluate the impact of manure application on the bacterial community and resistome of manured soils, Macedo et al. [106] investigated the soil communities of six dairy farms and found that Bacteroidetes, Proteobacteria, Verrucomicrobia, Actinobacteria, and Acidobacteria were the most abundant phyla. Differential abundance analysis showed that seven of the 30 most abundant families increased significantly after fertilization, including typical environmental bacteria *Burkholderiaceae*, *Chitinophagaceae*, and *Flavobacteriaceae*. Specific families increased either through the input of manure (e.g., *Dysgonomonadaceae*) or through enrichment after manuring (e.g., *Pseudomonadaceae*). These results suggest that applying organic fertilizer can significantly alter bacterial abundance.

To further understand how management modulates the soil microbiome, Jurburg et al. [107] surveyed the soil samples of 19 shade coffee plantations in Nicaraguan. On average, Proteobacteria, Verrucomicrobia, and Acidobacteria dominated the soil bacterial community in all samples. The relative abundance of candidate division WS3 was consistently higher in organically managed plots, while the relative abundance of Nitrospirae and Chloroflexi was higher in conventionally managed plots. Compared with bacterial communities, fungal communities were more variable across plots. The dominant phyla of fungal communities were Ascomycota, Zygomycota, and Basidiomycota. Moreover, organically managed plots had a higher relative abundance of Ascomycota, while Chytridiomycota was more abundant in conventionally managed plots. Their results show that soil bacterial and fungal communities were significantly altered by management.

Although the taxonomic resolution of soil studies is mostly restrained at the higher taxonomic level (e.g., phylum, class, or family) due to the complexity of soil microbial community, we can still observe the significant effects of different agricultural fertilization and management strategies on microbial communities besides climate and geography.

#### Landfill and leachate

In broad terms, landfills are extensive man‐made landscape features consisting of millions of milligrams of waste made up of artificial and natural organic materials, inorganic components, and buried local soils [108]. Materials piled up in landfills are challenging to degrade completely, resulting in the production of leachate [109]. Landfill sites are highly heterogeneous due to the substrate complexity, hence it has been considered a rich source of microbial diversity [110]. In the last few years, the microbes that mediate the biodegradation of discarded material have acquired substantial attention from the public [111]. Characterization of landfill microbiomes can also identify microbes with potential biodegradation capabilities [109]. Below we describe the general biological characteristics and composition of microorganisms in landfills.

To explore the structures of the bacterial communities in landfills, Kumar et al. [110] collected soil, leachate, and compost samples from different locations (heights and depths) at the landfill in Ahmedabad, India. A total of 2468 species, 793 genera, 278 families, 125 orders, and 58 classes were detected. Proteobacteria, Bacteroidetes, Firmicutes, and Actinobacteria were the main phyla in soil and compost samples. Firmicutes were the main phylum in leachate samples, followed by Actinobacteria and Proteobacteria. These results suggest that the relative abundance of bacterial community varied greatly between soil/compost and leachate.

Stamps et al. [109] investigated the diversity and composition of bacterial and archaeal populations in leachate from 19 nonhazardous landfills in 16 states of the United States. Numerous lineages of Proteobacteria (e.g., beta‐, delta‐, epsilon‐, and gammaproteobacteria) were most abundant. The researchers further divided the leachate samples into four main clades. The microbiome in clade A was mainly composed of Clostridia species. The microbiome in clade B was unique in the abundance of Campylobacterales species. Compared with other clades, the microbial communities of clade C showed systematic evolutionary diversity, including Chlorobi and members of candidate division OP9 in one landfill. Clade D included a larger population of candidate division OP3, Desulfobacterales, and Methylococcales. These findings suggest that landfills are a source of considerable bacterial and archaeal diversity and illustrate how leachate microbiomes are distinct among different landfills.

Moreover, the microbial composition of the surface soil could be changed substantially due to the migration of landfill leachate (LFL). Gu et al. [112] compared the microbial composition of uncontaminated soil and LFL contaminated soil from an unofficial landfill in China. They identified 63 phyla, 184 classes, 412 orders, 635 families, and 2200 species from all samples. The microbial diversity of soil in the contaminated area was lower than that of uncontaminated soil. The dominant phyla in uncontaminated soils included Proteobacteria, Chloroflexi, Actinobacteria, and Acidobacteria. In the contaminated soils, the predominant bacteria were Firmicutes, Proteobacteria, Chloroflexi, and Actinobacteria. Network analysis showed that *Bacillus*, *Clostridium*, and *Thermacetogenium* of the phylum Firmicutes were the keystone taxa and played a vital role in maintaining the stability of the soil ecosystem.

These studies demonstrate that landfill or leachate contamination can significantly change local soil microbial composition. Some microbes may have practical biodegrading abilities. Of note, microbes can be carried by air to further impact near and far human communities.

### Water

According to a report by the World Health Organization (WHO) in 2019, 1/3 of the world still does not have stable access to clean and safe water [113]. Modern technology has provided us with clean water supplies and wastewater treatment systems in more developed countries, but these systems created unique issues caused by pollutants. These pollutants inevitably affect surface water and associated ecosystems, as rivers, lakes, and oceans are both the input of drinking water supply systems and the output of wastewater treatment systems. Below we describe the main components of exposures from three types of water environments, surface water, drinking water distribution system (DWDS), and wastewater treatment plant (WWTP), recapitulating the utility cycle that natural water goes through.

#### Surface water

Surface water includes streams, lakes, rivers, and oceans and may also be referred to as blue water [114]. Alongside contributing to the most significant portion of human drinking water, surface water is also used for irrigation, livestock, industry, hydropower, wastewater treatment, and recreational purposes [115]. US Environmental Protection Agency (USEPA) recorded that approximately 68% of water provided to communities came from surface water [116]. According to United States Geological Survey (USGS) water‐use reports, surface water is considered freshwater when dissolved solids are below 1000 mg/L [117]. Microorganisms, including bacterioplankton and microeukaryotes, have received increasing attention as important components of aquatic ecosystems [118]. Below, we summarized the findings of several recently published studies as examples to illustrate biological exposures in surface water ecosystems.

The variations in surface water exposure composition can be attributed mainly to the temporal and spatial dimensions. A recent study investigated the microbiota dynamics in the community composition of a 1432‐km canal of the South‐to‐North Water Diversion Projects in China [119]. Along the canal, the phylum Cyanobacteria and Bacteroidetes showed a significant decrease in relative abundance, while two genera of Proteobacteria, *Candidatus*, *Methylopumilus*, and *Limnohabitans* had an increased abundance along the canal. In addition, seasonal variation was observed for specific bacterial and microeukaryotic lineages. Their results showed that seasonality could explain 36% of the microbial community variance, and 22% could be explained exclusively by environmental and spatial factors.

Moreover, anthropogenic input, physicochemical conditions, and hydrologic gradient also potentially influence surface water exposure components. Specifically, (1) Alexandra et al. [120] examined the microbial diversity in samples from the Kalamas River (Northwest Greece). This midsized river runs through farmland and receives urban sewage from a large city. They found that microbial human gut signals were more detectable than background freshwater and soil/runoff‐related signals, even tens of kilometers away from the city. (2) pH is one of the critical indicators of water physicochemical conditions. Krause et al. [121] performed acidification experiments on the bacterial community from the North Sea to explore the direct pH effects. They showed that small pH changes directly affected bacterial community composition and identified *Campylobacteraceae*, *Flavobacteriaceae*, and *Rhodobacteraceae* as phylogenetic groups responding notably to pH changes. (3) A recent study [122] characterized the taxonomic composition of bacterioplankton communities from 10 streams and rivers in Québec, spanning the whole hydrologic continuum. They found that decreasing bacterial richness and selective enrichment of Betaproteobacteria, Actinobacteria, and Cyanobacteria were associated with increasing distance from headwaters.

Reddington et al. [123] investigated the metagenomes of 11 rivers across three continents (Europe, North America, and Oceania) using MinION nanopore sequencing. The five most common bacterial phyla observed were Actinobacteria, Bacteroidetes, Cyanobacteria, Firmicutes, and Proteobacteria. The most common bacterial genera were *Acidovorax*, *Flavobacterium*, *Polaromonas*, *Polynucleobacter*, and *Streptomyces*. These microbes are the predominant drivers of water and soil ecosystem processes. The rivers also had other nonbacterial groups, including Apicomplexa (parasitic), Ascomycota (yeasts and molds), Arthropoda (insects and spiders), Bacillariophyta (diatoms), Basidiomycota (galls, mushrooms, smuts, and yeasts), Chlorophyta (*Chlamydomonas* and *Volvox*), Chordata (amphibians, birds, fishes, insectivores, and rodents), Cnidaria (Anthozoa and Hydrozoa), Nematoda (nematodes and roundworms), Protists (e.g., amoeba, ciliates, and flagellates), and Streptophyta (*Arabidopsis*, castor, corn, grape, mosses, *Populus*, rice, and wheat). In many cases, these observed taxa reflect the impact of upstream agricultural and urban activities.

Zhang et al. [124] studied the Ganges River microbial community and found that Proteobacteria and Actinobacteria were the most abundant phyla. At the same time, typical freshwater bacteria, such as Bacteroidetes, Betaproteobacteria, and Verrucomicrobia, were also detected. Ganges River was characterized by a high abundance of Gammaproteobacteria, which usually grow fast under conditions with enriched organic substrates, such as sewage lagoons. Notably, the Ganges River has also become a habitat for the populations of allochthonous bacteria, including WWTP‐associated *Candidatus Nitrospira defluvii* and *Thauera* sp. MZ1T; pathogens *Acinetobacter baumannii*, *Acinetobacter junii*, and *Pseudomonas aeruginosa*; antimicrobial‐producing *Rheinheimera* sp. A13L; *Pseudomonas putida*; and *Chthoniobacter flavus*. Abundant human gut‐associated microbes were also found in the Ganges River, including *Acinetobacter*, *Alistipes*, *Bacteroides*, *Caulobacter*, *Escherichia*, *Geobacter*, *Prevotella*, and *Oxalobacter* at the genus level.

Eraqi et al. [125] offered insights into the microbial composition of the Nile River. The community was dominated by the Actinobacteria, Cyanobacteria (mainly *Synechococcus*), and Proteobacteria (primarily *Comamonadaceae*). Among these dominant taxa, *Synechococcus* exhibited seasonal‐driven variation in relative abundance. Other taxa were predominantly rare across all seasons and locations, including genera implicated as pathogens, such as *Acinetobacter*, *Aeromonas*, and *Legionella*. In addition, comparisons with data on the freshwater microbiome in other world regions suggest that surface water communities in large rivers exhibit limited variation. These results showed striking stability in the Nile River microbiome community structure along the examined geographical urban sites and between the wet and dry seasons.

Ocean water is not a primary source of drinking water globally, but we come in close contact with ocean water through at least food, utility, and recreational means. The oceans have enormous biomass, measured in gigatons of carbon (GtC). About ~80% of the total marine biomass is mainly composed of animals (e.g., fish and crustaceans), protists (mainly eukaryotic microalgae and unicellular eukaryotes), and bacteria (e.g., photosynthetic cyanobacteria and heterotrophic bacteria) [126]. Two groups of bacteria were dominant and widespread [127]. One group, the SAR11 cluster, consists of tiny heterotrophic bacteria, which account for ~10% of the total bacterial biomass [128]. The other group comprises *Synechococcus* and *Prochlorococcus*, two ubiquitous genera belonging to the phylum Cyanobacteria. The total biomass of these two genera is estimated at ~15% of marine bacterial biomass.

In addition, some exotic species may invade the surface water and dominate the environment. Examples of aquatic bioinvasions are harmful algal bloom (HAB) or red tide, *Vibrio cholera, Cercopagis pengoi*, mitten crab, *Asterias amurensis*, and *Carcinus maenas* [129]. These invaders can disrupt complex ecosystems, reduce biodiversity, degrade habitats, and increase the impact on human health and the economy. For instance, red tide can be triggered by HABs as a natural phenomenon. Of more than 5000 microscopic algae species or phytoplankton that exist worldwide, about 300 species can cause red tides. One‐fourth of them is known to be harmful or toxic. Among these, Cyanobacteria, dinoflagellates, and diatoms are three main types of algae that cause HAB. Cyanobacteria species can bloom in freshwater lakes and rivers. Other algal species, including diatoms and dinoflagellates, commonly referred to as red tides, are found primarily in marine environments [129].

Biological exposures in surface water systems are immensely complicated and warrant more research on the topic. However, existing studies have demonstrated the consistent significant impact of anthropogenic activities, which can disrupt the surface water ecosystems to the detriment of all living organisms.

#### Drinking water and distribution systems

DWDS are complex water environments with multiple ecological niches supporting microbial growth [130]. Microorganisms are either planktonic cells suspended in a large amount of fluid or sessile cells embedded in the biofilms attached to pipe walls and other solid surfaces [131]. The microorganisms in biofilms comprise approximately 95% of the total biomass in a distribution system [132]. Previous findings indicate that the microbial community of drinking water is dominated by bacteria [130]. Archaea, fungi, viruses, algae, and protozoa (such as *Amoebas*) may also be present in DWDS, but their proportions are relatively small [133].

Several studies have highlighted the influence of specific characteristics on DWDS microbial community [130], including treatment strategies [134], distribution [135], process operations [136], hydraulic conditions [137], water age [138], residence time [139], and piping materials [138]. There are highly variable physicochemical interactions between different piping materials, dynamic hydraulics, and disinfection regimes [140]. Piping materials and hydraulic conditions affect the adhesion strength of pipelines, the volume of biofilms, and the microbial diversity of these ecosystems. DWDS microbial communities exhibit seasonal variations, as alpha diversity has a strong temporal trend associated with the temperature change [130].

Potgieter et al. [130] identified 60 bacterial phyla from a large, full‐scale DWDS in South Africa by 16S rRNA sequencing. Proteobacteria was the most dominant phylum in all samples. Further characterizations showed that the dominating groups were Alphaproteobacteria, Betaproteobacteria, Planctomycetes, and Gammaproteobacteria. Another meta‐analysis also showed that Proteobacteria was the dominant bacterial phylum regardless of whether disinfectant residues were present in the system [141]. Alpha‐ and beta‐proteobacteria accounted for more than 80% of proteobacterial sequences. Acidobacteria was the second most abundant phylum in the DWDS locations without residual disinfectant, and Actinobacteria were the second most prevalent phylum in disinfected systems.

More than 500 potential pathogens can be present in drinking water [132]. Pruden et al. [142] enumerated the plumbing pathogens of concern, including *Legionella pneumophila*, which causes Legionnaires' disease; *Mycobacterium avium*, which is associated with pulmonary diseases; *P. aeruginosa*, which is related to lung, urinary tract, and blood infections; and *Acanthamoeba*, which is associated with Acanthamoeba keratitis. Due to biofilm formation and disinfectant depletion, opportunistic pathogens, such as *Aeromonas*, *Legionella*, and *Mycobacteria*, can regrow in sterilized distribution systems [132, 143].

These studies demonstrate that while considered clean and safe, the DWDS microbial ecosystems can be easily enriched with potentially pathogenic microbes, especially considering the low background natural microbial diversity of the DWDS.

#### Wastewater treatment plant

Wastewater is a primary source of antibiotic‐resistant bacteria in the environment [144]. Public WWTPs receive a variety of anthropogenic antimicrobial and microbial contaminants, including antibiotics, fungicides, metals, and human pathogens [54]. The wastewater treatment process is designed to maximize the removal of pathogens, nutrients, and toxic compounds from wastewater before releasing it into the environment [144]. Microorganisms in bioreactors include bacteria, microeukaryotes, archaea, and viruses [145]. The composition of wastewater exposures depends on wastewater sources and a series of optional operations during treatment, for example, (1) influent composition. The types of wastewater include municipal, industrial, hospital, field runoff, and so forth. Different types of wastewater have different biological compositions. Among the eight WWTPs investigated by Wang et al. [146], ammonia‐oxidizing bacteria showed higher diversity in municipal WWTPs than in industrial or mixed WWTPs. (2) Process operation. Identical influents treated in different mains can result in differences in microbial community structures. For example, a WWTP processed the influent with an oxidation ditch (OD) and a membrane bioreactor (MBR) in parallel. Bacteroidetes was the most predominant phylum in OD samples, but the MBR samples were dominated by phylum Proteobacteria [147]. (3) Operational parameters. For example, the abundance of ammonia‐oxidizing bacteria is primarily affected by sludge retention time, while ammonia oxidation activity is mainly influenced by dissolved oxygen [148].

Several studies have investigated the microbial composition of wastewater bioreactor sediments, outlet sediments, and treated water [144, 149, 150]. Hameed et al. [149] monitored bacteria and archaea in two cascading digesters during the temperature‐phased anaerobic digestion (TPAD) process in municipal wastewater sludge obtained from Blue Plains Advanced WWTP. Twenty‐three phyla, 54 orders, 101 families, and 209 genera of bacteria were identified. Firmicutes was the most dominant phylum among all samples, followed by Bacteroidetes and Proteobacteria. Firmicutes is a common and highly diverse phylum that has been reported to occur during anaerobic digestion of sludge, such as chicken and cow manure, TPAD sludge, and activated sludge from various municipal WWTPs [149]. Bacteroidetes and Proteobacteria are the two most dominant bacterial phyla in the sludge obtained in the aerobic digestion stage of the bioreactor [150], indicating the difference in the microbial community between aerobic and anaerobic digestion. Furthermore, the class Clostridia is the most dominant among Firmicutes. From least to most, three orders of Clostridia were identified: Clostridiales, Thermoanaerobacterales, and Halanaerobiales. Two classes within the phylum Bacteroidetes, Bacteroidia and Flavobacteriia, were identified. Archaea communities also existed in the sediment samples of the anaerobic reactor, mainly composed of two classes, Methanomicrobia and Methanobacteria. Methanomicrobia (especially *Methanosarcinaceae*) constitute the majority of methanogenic communities, followed by Methanobacteria (mainly *Methanobacteriaceae* and *Methanomicrobiaceae*) from all samples.

Chu et al. [144] compared the bacterial community structure between WWTP effluents and corresponding sediment samples close to the effluents by inferring from the genetic composition. At the phylum level, Bacteroidetes and Firmicutes were dominant in effluent samples, while Actinobacteria, Bacteroidetes, Cyanobacteria, Firmicutes, and Nitrospirae were prevalent in the sediment samples. In addition, multiple polymerase chain reaction‐ and culture‐based studies have detected vancomycin‐resistant *Enterococcus*, methicillin‐resistant *Staphylococcus*, and cefazolin‐resistant *Enterobacteriaceae* in the biofilms of the final effluent, and clinically relevant antibiotic resistance genes (such as *CTX‐M*, *ampC*, *qnr*, and *NDM‐1*) [54, 151, 152, 153].

The wastewater treatment system has been a hotspot for research on antibiotics and metal resistance. The artificial environment promotes the exchange of microbial genetic materials, some of which will be released into a broad environment. Constant monitoring of the WWTP is needed to avoid further worsening impact on the dissemination and prevalence of multiresistant microbes in the environment.

### Disease‐related biological exposures

In daily activities, we are constantly in contact with biological exposures from all environmental sources; some can threaten our health. Pathogens can get into the air, water, and soil around us and invade our bodies through the common environmental exposure pathways. Importantly, human hosts inevitably become an amplifying source of dissemination as disease‐related microorganisms reproduce within the human body. These pathogens will be released into the immediate environment of a human living sphere and may transmit to another individual. Although we do not intend to write this review as a human‐centric depiction of the impact of the exposome, we use this opportunity to illustrate the effect of some biological exposures on human health.

Opportunistic pathogens are microbes that are not usually infectious to healthy people but may cause severe consequences in immunosuppressed patients or patients with other comorbid diseases, such as cystic fibrosis [154]. Most of the prevalent opportunistic pathogens belong to commensal bacteria. Antibiotics abuse can kill commensals without distinction and effectively selects commensal bacteria with more antibiotic resistance over time, increasing the incidence of infections that are insensitive to antibiotics treatments [155]. One study investigated the epidemiology of nosocomial bacterial colonization and infection in an acute rehabilitation unit. They found that vancomycin‐resistant *Enterococcus* and methicillin‐resistant *Staphylococcus aureus* (MRSA) were the most commonly identified colonizing organisms [156]. Notably, the exposome and microbiome of different body parts are tightly interconnected; exposure to one species in upper respiratory or oral systems can lead to exposures and infections in lower respiratory or gut systems, respectively, with often worse symptoms [157, 158]. Furthermore, it is plausible now that microbial exposures can even impact cancer progression [159].

As a primary focus in medical research, infectious, opportunistic, and multiple drug/antibiotic‐resistant pathogens have been studied extensively down to the mechanistic level. Mechanisms underlying opportunistic infections in immune‐compromised individuals have been investigated [160, 161]. For example, *L. pneumophila* is a ubiquitous opportunistic pathogen, the leading cause of legionellosis. In soil and aquatic systems, it can invade and colonize the interior cells of various protozoa. Under the protection of the host's biofilm, *L. pneumophila* can overcome environmental stresses (e.g., disinfection). Human infection by *L. pneumophila* occurs after inhaling aerosols containing the pathogen. Upon infection, *L. pneumophila* can enter and proliferate in macrophages in the alveoli. It mainly relies on the Dot/Icm type IV secretory system (a specialized protein transport system) to overcome the killing mechanisms of phagocytes. When a host cell is killed and ruptured, bacteria are released from it and infect other host cells, creating a new cycle of infection [162]. *C. difficile* is an intestinal pathogen that causes severe diarrhea and can even lead to death. *C. difficile* grows when exposed to primary bile acids in the gut. Without resistance from normal colonization, the pathogen colonizes the colon and produces toxins. These toxins can inhibit actin aggregation in host cells, leading to cell death [163].

Studying disease‐related biological exposures and their transmission routes in the exposome framework is of great importance. For example, during the initial stage of the pandemic, there was a debate on whether the viral pathogen is mainly transmitted based on contact or airborne, which greatly impacted the general opinion about wearing masks. For several months, contact spread was thought to be the main route by WHO [164]. Still, later research [165, 166, 167, 168] revealed that the airborne route is as critical if not more important than contact‐based transmissions, consistent with the highly efficient global spread of the virus. The airborn transmission route undoubtedly increases the risk of infection for society, which could lead to super spreading events [76]. On a related note, Jiang et al. [39] found that while only a few bacterial and fungal pathogens can be detected in the personal exposome at the low abundance level from time to time, exposures to bacterial and fungal opportunistic pathogens are nearly ubiquitous. Cissé et al. [169] reanalyzed the personal exposome data with a focus on *Pneumocystis jirovecii*, a well‐known fungal pathogen that causes pneumonia in immunocompromised patients, and showed that the infected individuals are likely to spread *P. jirovecii* in their personal “microbial clouds” continuously, a transmitting approach that was not described previously for the pathogen.

Short‐term biological exposures which lead to acute consequences have been scrutinized historically [170, 171]. However, the impact of long‐term exposure to countless known or unknown biological exposures is largely unknown. Scientists are just starting to recognize the effects of early‐life exposures on the development of immune systems, which can have a far‐reaching impact on immune‐ and psychosocial disorders in the later stage of life [172, 173, 174].

In summary, studying how pathogenic and opportunistic organisms distribute in our external and internal environments and how they evolve is crucial in controlling infectious and opportunistic diseases. The long‐term effects of biological exposure should not be underestimated.

## AN ATLAS OF THE ENVIRONMENTAL CHEMICAL EXPOSOME

Chemical exposures in the environmental exposome have been historically studied in the contexts of several different fields, including ecology [175], environmental toxicology [176], developmental biology [177], public health [178], atmospheric science [179], chemistry [180], and related industry [181]. Compared with the biological exposome, which is primarily nature‐derived, many chemical exposures are synthetic due to the ubiquitous use of human‐made chemicals in modern industry. More than 140,000 new chemicals and pesticides have been synthesized since 1950. Among which, the 5000 have become widely dispersed in the environment and are responsible for nearly universal human exposure [182]. Many chemicals can cause cancer or other chronic human health effects, adverse acute human health effects, and adverse environmental effects. A total of 775 chemicals and 33 chemical categories with at least one of these effects were listed by the Toxics Release Inventory Program of USEPA [183].

The impact of many anthropogenic chemicals on health has been investigated previously [184, 185]. However, the effects of chemicals of natural origin are severely underappreciated. Fungi, bacteria, and plants are nature's primary sources of chemical synthesis. Many chemicals may further react and transform the environment through biological and chemical means, leading to a more diverse collection of chemicals. Historically, the effects of some natural chemicals have been used for medical purposes, such as antibiotics, aspirin, and artemisinin, often without a complete understanding of the underlying mechanisms. Scientifically speaking, compared with biological exposures, constructing a comprehensive chemical database is even more daunting because each biological species can at least produce some unique compounds, and even a slight modification of the existing compounds could produce new compounds. A biochemical knowledge network called ATLASx predicted more than 5 million reactions and integrated nearly 2 million naturally and synthetically derived compounds [186].

**Figure 3:**
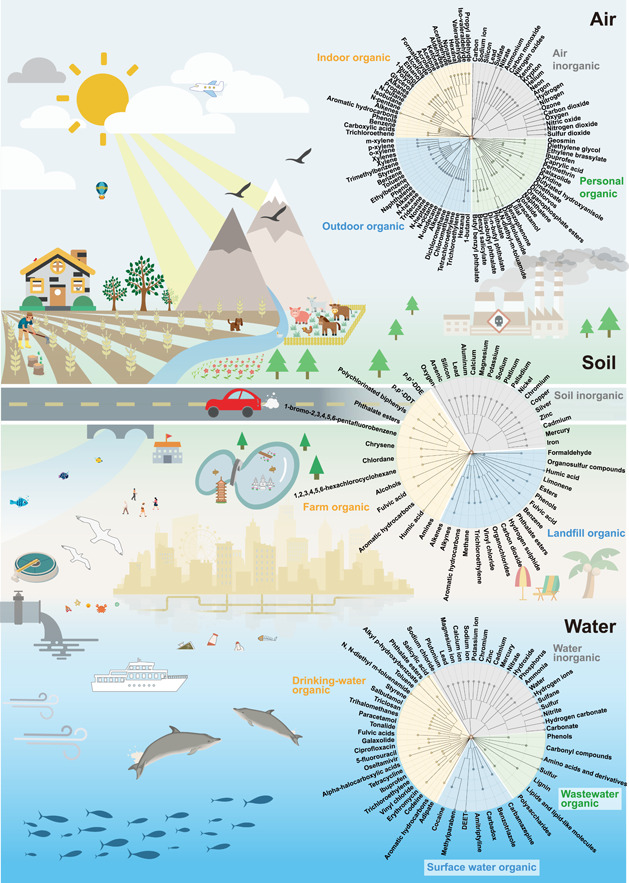
Different chemical exposures in air, soil, and water matrices. Summarized chemical exposures in different environmental matrices are represented in the form of assembled chemical taxonomic trees (circles on the right). Each circle is assembled by three or four taxonomic trees based on distinct subenvironments (represented by different colors within a circle). Note that inorganics for each air, soil, and water matrices are integrated and represented by one chemical taxonomic tree. The chemical taxonomy of summarized chemicals/chemical groups is generated by ClassyFire [187]. Trees are constructed using R package data.tree (version 1.0.0), and visualized using the online tool iTOL (https://itol.embl.de/) [63]. Potential sources of chemical exposures are also illustrated.

Similar to biological exposures, chemical exposures are structurally related and can be classified into superclasses and subclasses. ClassyFire [187] is a general classification system for small molecules based on their structures, whose chemical taxonomy consists of 11 ranks (Kingdom, SuperClass, Class, SubClass, etc.). The top level is Kingdom, which partitions compounds into two categories: organic and inorganic compounds. Below, we illustrate the representative inorganic matter for air, soil, and water matrices and the organic matter for each subcategory of these three matrices (Figure 3 and Supporting Information Table S2).

### Air

Air consists of nitrogen, oxygen, argon, carbon dioxide (CO_2_), neon, helium, krypton, hydrogen, and xenon [188]. Human activities can introduce additional gaseous/volatile compounds into the air [189]. These compounds have the opportunity to enter our bodies through skin‐contact or respiratory tract, thus adding complexity to the airborne chemical exposome. Specifically, USEPA has defined six criteria pollutants (PM, O_3_, nitrogen oxides, sulfur dioxide [SO_2_], carbon monoxide [CO], and lead [Pb]) as they can impact broad regions [190]. Together with inorganic compounds, some organic chemicals greatly expand pollutants into a larger group and are more harmful to human health. A list of 188 chemical exposures is defined as air poisons by USEPA because they can cause cancer or other serious health effects [191]. The main sources of these air poisons include vehicle emissions, factories, refineries, and power plants [192].

#### Inorganic matter

Inorganic air pollutants include some well‐known gaseous pollutants and PM in both indoor and outdoor environments. The inorganic gaseous pollutants include O_3_, CO_2_, CO, nitric oxide (NO), NO_2_, and SO_2_ [193]. These gases can form ionic substances after chemical reaction in the air and constitute PM with other elements, including ammonium (NH_4_^+^), nitrate (NO_3_^−^), sulfate (SO_4_^2−^), carbon, silicon, and sodium ion [194]. PM can be inhaled, especially PM2.5 (particles with an aerodynamic diameter less than 2.5 μm) can pass through the respiratory barrier into the circulatory system [195]. PM2.5 has been associated with increased morbidity and mortality of cardiopulmonary diseases [196, 197], occurrence and progression of diabetes mellitus [198], and kidney dysfunction [199].

Some population‐based studies have revealed the influence of specific chemicals or chemical groups on public health. For example, a study with 7134 participants collected by the National Health and Nutrition Examination Survey revealed that PM2.5 might increase the risk of cardiovascular disease for adults with metabolic syndrome (MetS) [200]. Another research called the Wuhan Chronic Disease Cohort study (WCDCS) recruited 10,253 residents to explore the effects of some air pollutants on MetS. Their results indicated that higher concentrations of PM10, PM2.5, and O_3_ were associated with higher MetS prevalence [201]. Besides, several elements and organic compounds can enter the geochemical cycle through atmospheric dust deposition and affect other environmental systems [202].

Pollutant concentrations are also seasonal. PM is typically higher in the eastern half of the United States from July through September, when sulfates form more readily from SO_2_ emissions from power plants in the region [203]. Huang et al. [204] monitored the outdoor PM2.5 density in three urban areas (Beijing, Tianjin, and Hebei) in China between 2013 and 2017. The average concentration of PM2.5 was 39 mg/m^3^ in spring and rose to 133 mg/m^3^ (5.3 times the WHO standards) by winter [205]. The concentration of pollutants in the air is related to industrialization processes. Hannah and Roser [206] described that the trends of SO_2_ emissions tend to follow an inverted‐U shape, first rising with industrialization before peaking and falling rapidly with further development. Indoor pollutant concentration is related to ventilation. In the case of stable outdoor PM concentrations, the air exchange rate significantly affects indoor PM concentrations. Generally, frequent air exchanges can reduce indoor PM concentrations if the outdoor air is relatively clean [207].

#### Organic matter

##### Indoor airborne exposome

Indoor air contains a complex mixture of VOCs and semi‐VOCs [208]. The generation and emission of traditional indoor air pollutants from combustion sources, building materials, furnishings, consumer products, personal care products, cleaning products, and gas‐phase reactions have been reviewed elsewhere [68, 209].

Cookstove smoke at home is the fourth leading cause of premature deaths globally [210]. Alves et al. [211] continuously measured the PM in a modern kitchen during the preparation of different Latin dishes, including fried horse mackerel, stuffed chicken, and grilled/fried pork strips. All cooking emissions were rich in propyl aldehyde, and there were higher levels of iso‐valeraldehyde and valeraldehyde during the frying of mackerel. PM10 accounted for more than 86% of the mass concentration of fine particles and contained alcohols, acids, plasticizers, alkyl esters, sterols, sugars, polyols, glycerol compounds, phenols, and so forth. Specifically, PM10 from grilled pork was highly toxic and posed a nonnegligible cancer risk.

VOC emissions from Chinese cooking may be more complicated. Wang et al. [212] sampled VOC emissions from kitchen pumping chimneys in Shanghai, China. It was found that 51.26 ± 23.87% of alkanes and 24.33 ± 11.69% of oxygenated VOC (OVOC) were dominant in cooking emissions. Cooking VOCs came mainly from heated oils and fatty acids. The heating of cooking oil can cause the decomposition of triglycerides into alkanes, alkenes, and OVOCs. Cooking fuels such as liquefied petroleum gas and natural gas were another source of alkanes, propane, *n*‐butane, and isobutane. The decomposition of fatty acids yields aldehydes. Barbequing was most likely to harm people's health because of the significant release of acetaldehyde, hexanal, and acrolein emissions. Overall, it is estimated that the total annual VOCs emissions of China's food industry in Shandong, Guangdong, and the entire country are estimated to be 5681, 6122, and 66,245 t/year [212].

The chemical properties of cooking VOCs vary by the Chinese cooking styles. Cheng et al. [213] collected VOCs from four Chinese cooking styles: barbecue, Hunan cuisine (more stir‐frying), home cooking, and Shandong cuisine (more boiling and steaming). The VOCs concentration and emission characteristics were analyzed. The results showed that the VOCs concentration of barbecue was the highest (3494 ± 1042 μg/m^3^), followed by Hunan cuisine (494.3 ± 288.8 μg/m^3^), home cooking (487.2 ± 139.5 μg/m^3^), and Shandong cuisine (257.5 ± 98.0 μg/m^3^). The abundance of alkane in emissions when cooking household dishes, Shandong dishes, and Hunan dishes is 59.4–63.8%; barbecue dishes' emissions are mainly alkane (34.7%) and olefin (39.9%).

Because outdoor exposures can influence indoor exposures, the location of buildings can also affect the composition of indoor chemical exposures. Villanueva et al. [214] investigated 32 VOCs in the classrooms of 18 schools in rural, urban, and industrial areas in Puertollano, Spain. Aldehydes (formaldehyde and hexanal) were the most abundant pollutants in all three regions. The concentration of benzene in industrial areas was significantly higher than in urban and rural areas, reflecting the contribution of nearby petrochemical plants to indoor air during the sampling period. Different VOCs had different primary sources, with benzene and *n*‐pentane originating from outdoor sources and aldehydes, terpenes, alkanes, and most aromatic hydrocarbons originating from indoor sources.

Similarly, the type and function of the building also introduce differences in indoor chemical exposure. Cometto‐Muñiz and Abraham [215] have investigated the indoor airborne chemicals of noncommercial (home and school) and commercial buildings. Compared with noncommercial buildings, the commercial buildings generally had higher concentrations of ketones, halogenated aliphatics, halogenated aromatics, and nonhalogenated aromatics. In contrast, aldehydes, carboxylic acids, cyclic aliphatics, and lineal aliphatics were higher in noncommercial buildings compared to commercial counterparts. In particular, there were 74 measured compounds found in both noncommercial and commercial environments. Of those, 32 were more abundant in noncommercial areas, 40 were more abundant in commercial areas, and the remaining two were present at similar levels. Of the chemicals higher in home and school environments, 1‐butanol, trichloroethene, and nonanal, were higher by more than five times. In contrast, 12 were higher by more than five times in commercial buildings, including acetone and ethanol.

In conclusion, human activities, mainly cooking, can lead to a sharp increase in indoor PM and VOC concentration. The building's geographical location—urban, suburban, rural, or industrial—determines the impact of outdoor air on indoor chemical exposure. And the type and function of the building can also cause differences in the composition of indoor chemical exposures. Given the ever‐increasing amount of time spent indoors, it is crucial to understand indoor chemical exposures and their impact on health [216].

##### Outdoor airborne exposome

Atmospheric pollutants include VOCs, such as benzene, toluene, ethylbenzene, and xylenes (collectively referred to as BTEX) [217]. The mean lifetimes of VOCs range from a few minutes to several months, allowing them to travel very long distances and expose to us via breathing or skin‐contact, posing a direct threat to human health. Moreover, as important precursors of ozone and secondary organic aerosols, VOCs have a significant impact on climate change and air quality [213]. VOCs participate in photochemical reactions to generate ozone, peroxyacetyl nitrate, and organic aerosols [218]. VOCs are emitted into the atmosphere from biological and anthropogenic sources. For example, methane (CH_4_) is generated from biological (natural wetlands and swamps) and anthropogenic sources (domestic livestock, landfills, and fossil fuel‐related emissions) [219].

Atmospheric VOCs have obvious seasonal and diurnal variations. Emission sources and meteorological conditions are the most important factors affecting the temporal distribution of the VOCs [218]. Guo et al. [220] investigated VOCs in the atmosphere of Hong Kong and found that VOC concentrations were seasonal. The concentrations of dichloromethane, xylene, and trimethylbenzene were slightly higher in summer than in winter. The concentrations of chloromethane, benzene, and tetrachloroethylene peaked in winter. Xie et al. [221] studied the total VOC content in Guangzhou. The results showed that the peak concentrations of alkanes and alkenes appeared at 8:00–10:00 in the morning and 18:00–22:00 in the evening, which were consistent with the traffic peak.

Outdoor chemical exposomes are distinct among cities, suburban, rural, and industrial areas. Researchers retrospected the airborne VOC levels of different areas (urban, suburban, and industrial) in Mexico and other emerging economies versus developed countries. Results showed that industrial and suburban areas reported higher VOC or BTEX levels due to fossil fuel burning and waste discharges. In large cities, VOC emissions are mainly from mobile sources. Although TEX levels were below the reference values, benzene was several times higher [217].

Industrial activity can greatly increase the concentration of concerned chemical exposures in the nearby outdoor air. Cometto‐Muñiz and Abraham's study [215] on air chemicals in outdoor environments comprised nonindustrial spaces (residential, urban, and semirural) and industrial areas (nearby a pig farm and an oil refinery). A total of 23 compounds were measured in both places, and they all had higher concentrations in the industrial spaces, with only ethanol as an exception. Among these, the concentrations of trichloroethylene and phenol were higher by more than 100 times; the concentrations of hexanal, 1‐butanol, *n*‐hexane, and tridecane were higher by more than 10 times; and the concentrations of benzene, ethylbenzene, nonane, *n*‐heptane, and *n*‐octane were 5–10 times higher. The remaining 11 compounds were elevated less than five times, including naphthalene, styrene, toluene, *n*‐undecane, *m*/*p*‐xylene, and *o*‐xylene. These results demonstrated the substantially higher concentrations of known pollutants in industrial areas.

Industrialization and vehicle emissions are major sources of outdoor harmful chemical exposure in suburban/rural and urban areas. The extreme long‐range and dynamic nature of outdoor airborne exposome makes it difficult to track but essential to study to get a comprehensive picture of the involved invisible risk factors. Importantly, the diversity and the extent of natural chemical exposures remain elusive.

##### Personal dynamic airborne exposome

The chemical categories related to daily life include chemicals in the plastics industry (e.g., phthalates and organophosphate esters [222]), personal care products (e.g., fragrances and ultraviolent [UV]‐blockers), pesticides (e.g., permethrin, *N*,*N*‐diethyl‐*m*‐toluamide), food industry (e.g., scents and butylated hydroxyanisole), and medicine (e.g., ibuprofen and paracetamol); some chemicals were assigned to multiple categories [41]. Many research projects have applied silicone wristbands to collect contact‐based chemical exposures [223, 224, 225, 226, 227]. For example, using silicone wristbands, Doherty et al. [41] assessed the multipollutant exposures during pregnancy. They deployed 255 wristbands and detected more than 1500 chemicals, among which 199 were identified in at least one wristband. On the basis of their results, the top 10 compounds presented most frequently included benzophenone, butyl benzyl phthalate, benzyl salicylate, diethyltoluamide (DEET), diisobutyl phthalate, di‐*n*‐butyl phthalate, ethylene brassylate, galaxolide, lilial, and tonalide. Another wearable sampler is the Fresh Air wristband [42]. It attaches a polydimethylsiloxane (PDMS) sorbent bar on a silicone wristband, which can passively collect and quantify the VOCs and polycyclic aromatic hydrocarbons (PAHs) in the air. The Fresh Air wristband has been deployed by the study of biomarkers of air pollutant exposure in Chinese people aged 60‐69 years (China BAPE) to systematically explore the associations between individual airborne exposures and adverse health outcomes [67]. They revealed that three types of exposures were highlighted based on elevated toxicity: dichlorvos from insecticides, naphthalene partly from mothballs, and polyaromatic hydrocarbons from multiple sources [228].

People are frequently exposed to thousands of expected and unexpected chemicals at specific locations. Jiang et al. [39] conducted a more comprehensive study on the personal exposome using wearable devices, and the chemical exposures were identified by liquid chromatography–mass spectrometry (LC–MS). According to their results, about 2900 chemicals were identified, and 972 were annotated. It is worth noting that chemicals detected are related to human production and life, including pesticides and carcinogens presented in everyday household products. For example, DEET, a commercially available insect repellent; omethoate, a pesticide; dimethoate oxide, an insecticide; phthalate, a plastic‐related chemical; pyridine, a common industrial organic solvent; and diethylene glycol (DEG), a carcinogen, were detected in the personal exposome. A cluster of 456 chemicals showed a sharp shift consistent with the seasonal transition in March, raising the possibility that exposure to these chemicals may be season driven. Interestingly, among a group of samples collected during rainy periods, geosmin (the “earthy” smell compound present when it rains), caprylic acid (commonly found in different types of disinfectants), and omethoate (a pesticide) were highly positively correlated with each other, suggesting that these chemicals can accumulate on the ground surfaces and be released during periods of rain. Notably, some compounds (e.g., DEET and DEG) may be enriched in different locations.

At the individual level, the exposed chemicals are of immense dynamics and variety and are tightly linked to health. We are just starting to investigate this frontier.

### Soil

Soil is a mixture of ~40–45% inorganic mineral matter, ~5% organic matter, ~25% gases, and ~25% liquids (v/v). The soil environment has several physical, biological, and chemical properties, and soil contaminants have both natural and synthetic origins [229, 230]. Over the past three centuries, anthropogenic activities such as industrialization, rapid urban development, and agricultural intensification represent the primary sources of soil pollution [231, 232, 233]. The most common entry routes of contaminants into the soil are direct application, atmospheric deposition, and application with irrigation water, rainwater, or river and lake sediments [234, 235, 236, 237]. Soil chemical exposure can occur via consumption or dermal contact [238, 239]. Unlike biological components, some chemical contaminants (e.g., heavy metals and persistent organic pollutants) cannot be chemically or biologically degraded, leading to the accumulation of pollutants in the soil environment. The residues of soil contaminants can be transferred and accumulated along the food chains and may pose short‐ and long‐term risks to human health [240]. In the following sections, we discuss the possible soil chemical exposome from the perspectives of inorganic and organic substances. We focus on the soil pollutants that may potentially impact human health.

#### Inorganic matter

Eight chemical elements comprise most of the inorganic mineral matter in soils, from most to least: oxygen, silicon, aluminum, iron, magnesium, calcium, sodium, and potassium [241]. A significant source of inorganic contamination is nitrogenous and phosphatic fertilizers, which affect soil properties, pollute runoff water, or sometimes escape into the atmosphere and affect the air quality [242]. Other inorganic pesticide ingredients may also be introduced into the farmland, including inorganic salts such as copper sulfate and ferrous sulfate, lime, sulfur, arsenic, cyanide, and mercury [243, 244]. Heavy metals are also present in the soil, including Pb, arsenic (As), mercury (Hg), cadmium (Cd), zinc (Zn), silver (Ag), copper (Cu), ferrum (Fe), chromium (Cr), nickel (Ni), palladium (Pd), and platinum (Pt) [245]. Some heavy metals (e.g., Cu and Zn) are essential nutrients at low concentrations, while some have toxic effects on both ecosystems and humans at higher concentrations [246]. Nevertheless, some trace elements in soils strongly influence the healthy growth of plants and the animals that graze on them [247]. Besides farmlands, landfills are also pools of heavy metals. Landfills of municipal solid waste release numerous pollutants to the environment via LFL or landfill gas (LFG) [248]. The infiltration of LFL is the leading cause of soil, groundwater, and surface water pollution.

#### Organic matter

##### Farm and rhizosphere

Plant degradation and microbial metabolism produce natural organic matter in the soil, including small molecules, such as organic acids, sugars, amines, and alcohols, and large molecules such as fulvic acid, humic acid, humin, and extracellular secretion. Soil organic matter contributes to soil aggregation, nutrient exchange, moisture retention, compaction reduction, and serves as a reservoir for crop nutrients [249]. Agricultural soil contamination is ubiquitous worldwide due to the long‐term application of fertilizers, pesticides, plastic film, wastewater irrigation, sewage application, and other human activities. The accumulation of pollutants in agricultural soil may harm soil ecology, plant growth, and human health. Several organic contaminants, for example, phthalate esters, PAHs, polychlorinated biphenyls, and organochlorine pesticides (OCPs) are highly toxic, bioaccumulative, and persistent in soil environments [250, 251]. According to Sun et al. [240], more than 20 kinds of OCPs and various PAHs can be present in Chinese farmlands. Of these OCPs, *p*,*p*′‐dichlorodiphenyldichloroethylene had the highest concentration, followed by *p*,*p*′‐dichlorodiphenyltrichloroethane (DDT) and chlordane. The average concentrations of DDTs and hexachlorocyclohexanes are 41.6 ± 57.2 and 11.4 ± 18.2 ng/g, respectively, which is comparable to those reported in Romania and Germany [252, 253]. The average concentration of PAH was 772 ± 895 ng/g. Benzo[*b*]fluoranthene and chrysene were the most abundant carcinogenic PAHs in the farmland. Due to their high hydrophobicity, PAHs are mainly produced by combustion processes and tend to be retained in the soil [254]. The USEPA has determined 16 PAHs on the priority control list [255]. As the contaminants in the agricultural soil are directly connected to the dining tables, a complete understanding of farm soil exposome is becoming an important goal in the field.

##### Landfill and leachate

The organic components of LFL are mainly dissolved organic matters (DOMs), often measured as chemical oxygen demand (COD) or total organic carbon (TOC), refractory compounds such as fulvic‐like and humic‐like compounds, and volatile fatty acids. Furthermore, the various studies showed the presence of LFL organic pollutants from both biogenic and xenobiotic origins [256]. Xenobiotic organic compounds originating from household or industrial chemicals include a variety of chlorinated aliphatics, pesticides, plasticizers, and surfactants, which have been frequently detected in the LFL [257].

Swati et al. [258] evaluated the soil from three municipal solid waste landfill sites in Delhi, India. Persistent organic contaminants, such as benzene derivatives, halogenated aliphatic compounds, phthalates, and PAHs were detected by gas chromatography–mass spectrometry (GC–MS). The authors discussed that although the low concentration of PAHs (192–348 µg/kg in total) in soil organic extract only had negligible carcinogenicity, the organic pollutant mixture in the soil was toxic enough to affect human health due to the synergistic or additive effects of chemicals.

In addition, as a major pathway for releasing pollutants from landfills, LFG emissions significantly influence local and regional air quality [259]. LFGs are mainly composed of CH_4_ (50–60% v/v), CO_2_ (20–40% v/v), and trace gases, and they can be generated by sludge degradation by microbes. The nonmethane VOCs (e.g., benzene, hydrogen sulfide, trichloroethylene, and vinyl chloride) in trace quantity (about 1% v/v) also belong to LFG. Several toxic VOCs with high volatility and low solubility can also present in LFG. VOC from landfills is estimated to account for 10% of total VOC emissions in the United States. In addition, pollution‐related aerosols can be produced by some unsaturated VOCs, such as alkenes and alkynes. Some VOCs (e.g., alkylbenzenes, esters, organosulfur compounds, and limonene) have intense odor. Other VOCs with benzene rings (e.g., benzene and formaldehyde) were considered carcinogenic to landfill workers and nearby residents [260].

The unpleasant and nauseating smell is the typical impression of landfill and leachate. The decomposing nature of this artificial type of land makes it a complicated blend of natural and anthropogenic chemicals, some of which require strict control.

### Water

Water is the medium for a wealth of organic and inorganic chemicals of both synthetic and natural origins. The concentrations of chemicals in any water body can quickly change due to evaporation or precipitation. Chemicals in the water exposome can also freely exchange with soil and air exposomes. Understanding the nature of the chemical exposome in the water has been a primary objective for scientists even before discovering microbes.

#### Inorganic matter

Many inorganic ingredients are common in surface water, drinking water, and wastewater. These inorganic constituents include hydrogen ions, hydroxyl and bicarbonate ions, chlorides, nitrogen, phosphorus, sulfur, and heavy metals [261]. The concentration of hydrogen irons reflects the acidity of water. Alkalinity is mainly determined by hydroxyl and bicarbonate ions caused by dissolved compounds in soil, such as the carbonate and bicarbonate of calcium, potassium, magnesium, and sodium. As a measure of acidity or alkalinity, the pH of water drives many chemical reactions in water environments and living organisms. Chloride is one of the main inorganic components in water. Nitrogen exists in the form of organic nitrogen, nitrite (NO_2_^−^), nitrate (NO_3_^−^), or ammonia (NH_3_) in water. NH_3_ exists naturally in wastewater and is produced mainly through the deoxidation of organic nitrogen compounds and urea hydrolysis. NO_2_^−^ is an intermediate oxidation state of nitrogen and can enter water systems by being used as corrosion inhibitors in industrial applications. NO_3_^−^ is derived from the oxidation of ammonia. Phosphorus, mainly in the form of phosphate, is ubiquitous in wastewater and essential for all living organisms. Municipal wastewater may contain 10–20 mg/L of phosphorus, mostly from detergents. Reducing the input of phosphorus into the water can control the eutrophication issue. Sulfur is essential for protein synthesis and is released upon degradation. Sulfate ions are naturally present in many water supply systems and wastewater. Sulfates are biologically reduced to sulfides, which can form hydrogen sulfide (H_2_S) with the combination of hydrogen. At higher concentrations, H_2_S is a deadly toxin. Heavy metals, including Cd, Hg, Zn, Cr, Pb, and Pu, are industrial effluents' main toxic substances. The excessive presence of any of these metals can interfere with many beneficial water uses [261].

#### Organic matter

##### Surface water

DOM is ubiquitous in aquatic systems, constituting one of the largest dynamic reservoirs of organic carbon on the Earth [262]. DOM concentrations measured as organic carbon are reported as 1–10 mg/L in rivers and 1–50 mg/L in lakes [263]. In the natural environment, DOMs play multiple vital roles as a nutrient source for aquatic organisms, a photosensitizer for anthropogenic compounds, and a chelating agent for trace metals. DOM is heterogeneous, making it difficult to determine its composition, so it is hard to isolate representative portions for the downstream molecular analysis [262].

The marine environment has a large amount of DOM, the aggregate carbon content of which is comparable to that of the atmosphere. The organic matters in the ocean are typically differentiated by their sizes, that is, whether they can pass through the filter with pore sizes between 0.2 and 0.7 μm. DOMs refer to the substances passed through the filter. In contrast, substances that retain on the filter are termed particulate organic matters (POMs) [267]. This classification is artificial but somewhat related to biogeochemical consequences. DOM is generally soluble in water, while POM can be deposited on the sea floor or suspended in marine water. Thus, DOM can remain in the water longer than POM. Aside from containing a few viruses and small prokaryotes, DOM is almost lifeless. POM includes a small portion of living biomass, such as phytoplankton, and a large portion of detritus, such as dead cells. Proteins (∼45%), carbohydrates (∼25%), lipids (∼17%), nucleic acids (∼12%), and pigments (∼2%) are the main substances constituting the living biomass of POM [267].

Many studies have focused on the pollution of rivers and lakes by human‐related activities and characterized the dissolved organic components. Minnesota Pollution Control Agency (MPCA) confirmed that many unregulated chemicals end up in the lakes and rivers of Minnesota [265, 266, 267]. Chemicals associated with medicines and personal care products have been detected, many of which interfere with the function of hormones in animals and humans. In one study [265], MPCA analyzed 125 chemicals in 50 randomly selected lakes in Minnesota. Commonly used chemicals were widely distributed in Minnesota lakes, including cocaine, the antibiotic carbadox, and the antidepressant amitriptyline. The insect repellent DEET was the most frequently detected compound. Another study [266] identified 18 compounds at 150 river locations selected randomly in Minnesota. Several personal care products and pharmaceuticals were present in these compounds. Parabens were commonly found, with methylparaben detected in more than 30% of the samples. Parabens is a family of chemicals widely used as preservatives for food and cosmetics. A breakdown product of the corrosion inhibitor benzotriazole was present in 12% of the samples. Carbamazepine, a component of medication to treat attention deficit hyperactivity disorder, and several antidepressants were also found.

The red tide can produce toxins that can destroy the aquatic ecosystem, affect the survival of marine animals, and even directly or indirectly affect human health. Some red tide species like dinoflagellate *Gymnodinium breve* can produce neurotoxins (e.g., dinotoxins [268]). It is a group of well‐known toxins that can paralyze the central nervous system of fish [128]. Some phytoplankton species produce polyunsaturated fatty acids and galactolipids, which lyse blood cells. Some algae also produce these hemolytic compounds and neurotoxins, and exposure to these chemicals can dramatically lower the fish's heart rate, leading to reduced blood flow and a lack of oxygen. Swimming or contact with the sea can potentially expose to these toxins directly, causing respiratory issues or skin irritation. Eating shellfish contaminated with red tide toxin can lead to human poisoning indirectly [129].

Surface water is filled with DOM and POM due to natural and anthropogenic activities, most of which are still unknown now, but some already show significant toxic effects on organisms.

##### Drinking water and distribution systems

Natural organics in drinking water include allochthonous organics mainly composed of humic acids and fulvic acids (which are more hydrophobic), and autochthonous organics consisting of carbohydrates and proteins (which are produced in water bodies and are more hydrophilic) [269]. More importantly, organic pollution in drinking water is often attributed to improper treatment and application of various municipal, agricultural and industrial processes, resulting in the contamination of drinking water by synthetic organic chemicals [270]. These chemicals include VOCs (e.g., toluene, styrene, trichloroethylene, and vinyl chloride), drugs (e.g., erythromycin, tetracycline, paracetamol, ibuprofen, and chemotherapy drugs, such as ifosfamide and 5‐fluorouracil), industrial compounds (e.g., chlorinated solvents, hydrocarbons, and petroleum), personal care products (e.g., DEET, alkyl *p*‐hydroxybenzoate, and triclosan), synthetic musks (e.g., tonalide and galaxolide), plasticizers, flame retardants, and surfactants. In many cases, they are carcinogenic endocrine disruptors.

In addition, disinfection byproducts can be produced when ingredients used to disinfect drinking water react with DOM [271]. For example, a survey observed trihalomethane and haloacetic acid in drinking water, which have potential reproductive, carcinogenic, and mutagenic effects. However, the health risks from disinfection byproducts are minimal compared with the risks associated with inadequate disinfection [270].

Chemicals in drinking water are always of special concern as no life can survive without timely water intake. Although people in developed countries do not normally worry about contaminants in drinking water, situations in developing countries warrant close monitoring.

##### Wastewater treatment plant

DOM plays a vital role in ecosystem processes and is the main removal and limiting factor for wastewater recycling and reuse [272]. The composition of DOM in wastewater depends on the type of wastewater (municipal, industrial, hospital, field runoff, etc.) and the nature of the treatment process used in WWTP. DOM in wastewater is a complex and heterogeneous mixture of polysaccharides, amino acids/peptides/proteins, lipids, nucleic acids, soluble microbial products, and anthropogenic organic chemicals. A study showed that DOM accounts for 82.6‐86.6% of TOC and 78.1–86.5% of total COD in WWTPs effluent. Anthropogenic compounds in wastewater include fungicides, industrial chemicals, medicines, personal care products, pesticides, and surfactants [273]. With wastewater processing, there is a wide range of bioactive transformation products, disinfection byproducts, intermediates, and metabolites. Some of these compounds can be dangerous even at low concentrations and are not expected to be released into environments.

DOM has a variety of functional groups, such as carbonyl, carboxyl, methoxyl, hydroxyl, and phenolic functional groups [272]. Maizel et al. [274] assessed DOM in the Nine Springs WWTP in Madison, Wisconsin, using UV–visible spectroscopy and Fourier transform ion cyclotron resonance MS. In total, 2106–3013 chemicals were identified in each sample in negative mode, while 815–1949 were identified in positive mode. Effluent organics generally contain recalcitrant organics, such as synthetic organic compounds produced during household and disinfection, lignin compounds from drinking water, and soluble microbial products from biological processes. Due to the presence of amino acids, detergents, pharmaceuticals, and surfactants, the sulfur content in wastewater is generally higher than in natural water.

Similar to landfills, the WWTP is another synthetic center of chemicals as the substrates and microbes are abundant for reactions to happen. Some of these chemicals are toxic and more efforts are needed to contain the spread of dangerous chemicals, even at low concentrations, to the natural water systems.

### Disease‐related chemical exposures

According to global statistics, 4.9 million deaths (8.3% of total) and 86 million disability‐adjusted life years (DALYs; 5.7% of total) can attribute to environmental exposures in 2004 [275]. This is more than the disease burden of all types of cancers (5.1% of all DALYs). Harmful chemical exposures may have short‐term or long‐term effects on human health [276].

Short‐term effects have a relatively quick onset, usually occurring minutes to days after brief exposure to relatively high levels of harmful chemicals. More than 2 million people suffer some types of poisoning each year in the United States. Prescription, over‐the‐counter, and illicit drugs are common sources of severe poisoning and poisoning‐related deaths. Other common poisons include gases (e.g., carbon monoxide), household products, agricultural products, heavy metals (e.g., iron and lead), vitamins, animal venom, and so on [277]. At the molecular level, a study observed that 3449 exosome mRNAs, 58 serum proteins, and 128 serum metabolites of participants were significantly changed after 4‐hour exposure to traffic‐related air pollution, involving dozens of regulatory pathways, such as growth hormone signaling, adrenomedullin signaling, and arachidonic acid metabolism [278].

Long‐term effects persist (or repeatedly occur) over an extended period. Repeated exposure to low concentrations of certain chemicals over a period of years can pose a potential long‐term risk [276]. A large number of international databases, organizations, and systematic reviews have studied and reviewed the burden of disease caused by chemical exposure [1]. Many epidemiological studies and major reviews have integrated exposure datasets with clinical information, for example, occupational asbestos and diesel exhaust exposure and lung cancer; occupational exposure to methanol and leukemia; secondhand smoke and trachea, bronchi, and lung cancer; arsenic exposure in drinking water and bladder cancer, kidney cancer, peripheral neuropathy, and red blood cell destruction; outdoor air pollution and cardiopulmonary diseases. However, the true impact of chemical exposure on health has still been dramatically underestimated [275]. Because many known chemicals of concern have not been considered, thousands of compounds recognized as safe have not been subjected to rigorous scientific testing [37].

It is difficult to assign specific exposures to certain diseases due to the delayed or subclinical health effects, such as cancer, cardiovascular disease, or certain neurological diseases. Thus, the burden of disease caused by currently known chemicals is large, while the unknown burden is most definitely considerable but difficult to estimate. Further investigations on chemical exposures and the effects of chemicals on population health are essential for taking targeted measures to limit exposure to harmful chemicals and ease the chemical‐induced global burden of various diseases.

## AN ATLAS OF THE ENVIRONMENTAL PHYSICAL EXPOSOME

The impact of physical exposome on human health is highly intuitive and broadly recognized by the scientific community and the public. For example, light, electromagnetic radiation of shorter wavelengths than visible light (UV light and seldomly more dangerous X‐ray or even Gamma‐ray), noise, force, and temperature have been subjects of extensive research.

Light exposure can have various effects on the health and mood of humans [279] and is also related to circadian rhythm patterns discovered in all types of organisms [280], although the strict dependence of circadian rhythms on light is controversial [281]. Excessive exposure to the light emitted from electronic devices, especially late at night, would lead to sleep issues and even insomnia [282]. As a part of the natural daylight spectrum, blue light can stimulate brain activity and help people stay awake and focused by suppressing the secretion of melatonin, a hormone that influences circadian body rhythms [283]. Recently, it has been shown in human and animal studies that exposure to sunlight can prevent myopia by inhibiting irregular axial ocular growth [284, 285, 286]. On the other hand, exposure to short‐wave UV light, X‐rays, and even Gamma rays can damage the organisms at the tissue, cellular, molecular, and DNA levels, leading to cancer and other types of severe diseases [287, 288, 289, 290].

Noise pollution from construction, traffic, aircraft, and your neighbors could impact millions of people daily [291]. Prolonged noise exposure can lead to hearing loss, high blood pressure, heart disease, sleep disturbances, and mental issues. According to the National Institute for Occupational Safety & Health (NIOSH), about 30 million workers are exposed to hazardous sound levels at work in the United States alone. Industries that are more impacted include agriculture, construction, manufacturing, military, mining, transportation, and utilities. According to WHO's findings, noise is the second largest environmental cause of health problems, only ranked after the impact of air pollution described earlier. However, more people overlook the adverse impact of noise pollution compared with air pollution [292], even though noise pollution can immediately and drastically impact life quality. In addition, human‐sourced noise also affects the welfare of wildlife in the sky, on the land, and deep in the ocean.

Excessive physical force inflicted by hazardous working environments, postures, and sports activities can lead to acute and chronic consequences for the well‐being of humans. Notably, some industries may put their workers in danger of being exposed to excessive physical force. Apart from acute physical injuries, inflammation, and long‐term pain, which are expected outcomes of exposure to excessive physical force, psychological damages often overlooked can also be tremendous and manifest in anxiety, depression, posttraumatic stress disorder, and even suicidal behaviors [293]. More research on the long‐term impact of excessive physical force is ongoing.

Drastic temperature variation is another leading cause of diseases or even death globally. Temperature variations due to activity, climate, and working environment can all impact the health state of individuals. Climate change has led to increasingly common heat waves. Excessive heatwaves, defined by a sudden increase in outdoor temperature over an extended period, have a wide range of physiological impacts on populations and can even lead to premature death and disability in predisposed individuals. Excessive heat can lead to heatstroke, heat cramp, hyperthermia, and worsening cardiovascular, respiratory, and cerebrovascular diseases [294].

Similar to excessive heat, exposure to frigid temperatures can lead to various health issues. Cold weather acts as a vasoconstrictor; a drop in temperature increases blood pressure and places more strain on the heart. As a result, there is an increase in heart attacks for populations with cardiovascular diseases. Cold and dry weather also damages the skin by sucking out moisture, leading to irritations, redness, frostnip, frostbite, and trench foot. Prolonged exposure to cold can also lead to asthma, arthritis, suppressed immune response as the body tries to conserve energy, hypothermia, and eventually death [295]. Intriguingly, exposure to cold temperatures in controlled and specific ways seems to confer certain health benefits. A recent study [296] found that indoor exposure to mildly cold or warm temperatures outside the so‐called comfort zone of 21–22°C can significantly increase metabolism. Ten days of intermittent cold spells for type 2 diabetes patients can improve insulin sensitivity by more than 40%, comparable with the best pharmaceutical treatments currently in use. Whole‐body cryotherapy has become increasingly studied and has shown positive effects on muscle soreness and decreases the recovery time after exercise [297]. In competitive sports, whole‐body cryotherapy in a cryochamber is used to treat athletes by placing individuals in a −184°C chamber for 2–3 min, which is supposed to stimulate a robust circulatory response throughout the entire body quickly. Once out of the cryochamber, the athletes continue doing cardio exercises to rewarm the body. More research needs to be dedicated to investigating the potential benefits of cryotherapy.

The summarized effects of physical exposures on health are by no means complete, but we hope to integrate the physical exposures under the comprehensive exposome framework as vast amounts of related research are available on the topic. More importantly, the physical exposome can often greatly influence biological and chemical exposomes, which we will discuss in the following section.

## THE INTERWEAVING BIOLOGICAL, CHEMICAL, AND PHYSICAL EXPOSOMES

**Figure 4:**
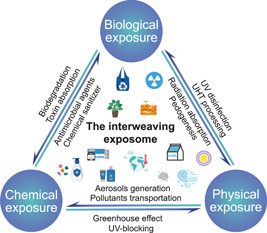
Interweaving biological, chemical, and physical exposomes. Interactions between exposures in three major domains of the environmental exposomes are demonstrated. The texts by the arrows illustrate some of the interactions closely related to humans. UHT, ultrahigh‐temperature processing; UV, ultraviolent.

Different components of the exposomes are constantly interacting with each other. Under the general exposome framework, the traditional environmental factors, such as temperature, humidity, pH, and salinity, are also considered parts of the exposome. Wind and monsoon can bring biological and chemical exposures to a great distance. Bacteria facilitate numerous physical and biochemical alterations or reactions in soils and thereby directly or indirectly support the development of plants. From this perspective, the influence of environmental factors on exposomes can be viewed as interactions among physical, biological, and chemical exposomes. Unraveling these intertwined interactions with concerted efforts from interdisciplinary fields will be essential to constructing the interweaving exposome framework in the future (Figure 4). Below, we discuss some of the well‐known interactions among these three main domains of environmental/external exposomes.

### Biological to chemical

All organisms are highly efficient factories of chemical reactions and consume, produce, or change the properties of chemical exposures. Microbes, for example, can facilitate a variety of chemical reactions to absorb or decompose materials. Humans have been taking advantage of microbes in the biodegradation of sewage or landfill treatment.

It is well accepted that microbes and plants play a huge role in metabolizing and producing many chemical exposures in the environment. For example, microbes play a role in structuring the chemistry and emissions of kitchen sinks and showers, producing microbial VOCs that are mainly related to fatty acid processing [298]. Some plants can also absorb some toxic substances, which are used to improve the indoor environment [299]. Boston fern (*Nephrolepis exaltata*) is considered the most effective plant in removing formaldehyde [300]. Golden pothos (*Epipremnum aureum*) can remove ammonia, formaldehyde, and acetone from indoor air [301]. It is recommended that plants and associated soil microbes can be used to reduce trace air pollutants in indoor environments [302].

Some chemical processes mediated by soil microbiome are critical to maintaining soil element cycling and properties. These chemical processes include ammonification (e.g., *Bacillus* and *Pseudomonas*), nitrification (e.g., *Nitrosomonas* and *Nitrobacter*), denitrification (e.g., *Achromobacter*, *Pseudomonas*, *Bacillus*, and *Micrococcus*), and nitrogen fixation (symbiotic *Rhizobium*, *Bradyrhizobium*, etc., nonsymbiotic *Azotobacter*, *Beijerinckia*, etc.). Actinomycetes can produce several secondary metabolites, such as antibiotics (e.g., streptomycin) and geosmin, responsible for the “earthy” smell after soil plowing. Actinomycetes are important in forming stable humus, which enhances soil structure, improves soil nutrient storage, and increases water retention in soil. The most commonly encountered soil actinomycetes belong to *Nocardia* and *Streptomyces* genera [303].

As the main force of decomposers, many microorganisms, such as *Bacillus*, *Achromobacter*, *Cellulomonas*, *Clostridium*, and *Methanococcus*, can decompose cellulose or even plastics [304]. Actinomycetes decompose a wide range of substances, but they are critical in degrading recalcitrant substances, such as chitin, lignin, keratin, and cellulose [303]. Microorganisms can also solubilize heavy metal pollutants by direct bacterial processes or interactions with metabolic products. It can be used in situ or ex situ to help remove the pollutants from soils [303]. Microorganisms are also widely used in wastewater treatment processes by their virtue of chemical reactions. *Truepera*, *Paracoccus*, and *Denitratisoma* were found to carry out denitrification to remove nitrogen. *Nitrospira*, *Thauera*, and *Dechloromonas* are the most abundant microbial genera in the anaerobic–anoxic–aerobic sludge. Further, *Nitrosomonas*, *Nitrospira*, and *Nitrobacter* have been identified as the key taxa for nitrite oxidation [305].

### Chemical to biological

Compounds provide essential nutrients for living organisms or mediate signal exchange between microorganisms. Chemical exposures can disturb the stability of biological communities. For example, soil fertilization in agriculture can alter the microbial community. Phytocides, insecticides, and antibiotics do great jobs at killing different kinds of organisms.

Heavy metals are well‐known environmental pollutants because of their toxicity, long life in environments, and ability to accumulate in organisms. Microorganisms are the first biota that undergoes direct and indirect impacts of heavy metals. Some metals (e.g., Fe, Zn, Cu, Ni, and cobalt [Co]) are vital for many microbial activities occurring at low concentrations. These metals are often involved in the metabolism and redox processes. Metals facilitate secondary metabolism in bacteria and fungi. For example, chromium is known to have a stimulatory effect on both actinorhodin production and the growth yield of the model actinomycete *Streptomyces coelicolor*. However, high concentrations of heavy metals may have inhibitory or even toxic effects on living organisms [303].

Humans have been using chemicals to disinfect drinking water and prevent food spoilage. Disinfection exhibits systematic impacts on the drinking water microbiome. Compared with unsterilized systems, microbiota in sterilized drinking water showed lower structural and functional diversity and variability [306]. Different disinfection strategies cause drastic changes in microbial communities. Alphaproteobacteria are generally dominant in chlorinated and chloraminated water, while Betaproteobacteria has increased abundance in chloraminated water but not in chlorinated water [307]. Chemical additives have been widely used to prevent the survival and proliferation of microorganisms in food. Acidifiers, organic acids, and *p*‐hydroxybenzoates are some of the common antibacterial agents [308]. Some natural substances such as plant essential oils and extracts also have antibacterial properties. Bacteriocins are produced by various microorganisms, some of which have shown significant antibacterial potential, and as the effective application of a natural barrier against food corruption [309].

The effects of chemical exposures on biological exposures are also exploited in personal care and medicine. Broad‐spectrum antibacterial agents, such as triclosan and triclocarban, are widely used in personal care products, including hand sanitizers, shampoos, body washes, and cosmetics [310]. Antibiotic therapy is typically used to target specific pathogenic skin colonizing bacteria, such as MRSA or Group A *Streptococcus* [311]. Povidone‐iodine is a water‐soluble compound consisting of the molecule iodine and polyvinylpyrrolidone. It works by releasing iodine that kills prokaryotic and eukaryotic cells through the oxidation of membrane compounds and lipid iodization [312]. Chlorhexidine's mechanism of action involves membrane disruption, leading to leakage and ultimately cell death. Ethanol acts as a bactericidal agent by dissolving lipid membranes and denaturing proteins.

### Biological to physical

Biological exposures rarely directly affect physical exposures and usually exert their impact through chemical reactions or substances. For example, the activity of humans and livestock, such as cows, chickens, and pigs contribute to global warming through the production of greenhouse gases (e.g., methane) at the global level [313]. Current estimates suggest that 90–95% of the methane released into the atmosphere is of biological origin and is entirely the result of microbial activity. The process of biomethane production is called methanogenesis mediated by the methanogens [314].

Living organisms can regulate the humidity and temperature of the environment. Plants in outdoor or indoor environments contribute to the elevation of moisture and temperature through respiration activity. Tropical forests can have a localized cooling effect by increasing humidity and promoting wind currents through transpiration. In addition, the shade of the forest canopy causes a significant reduction in temperature relative to areas exposed to direct sunlight. One of the biggest complaints of local people after deforestation is the rise in local temperatures [315].

Soil microorganisms and animals (e.g., earthworms and termites) can produce a large number of water‐stable microaggregates in soil, reducing soil bulk density and improving soil structure [316]. Both bacteria and fungi secrete sticky, polysaccharide slime that binds soil particles together to form aggregates. These aggregates can stabilize water action for several months and help prevent soil dispersion. The root systems of plants and fungal hyphae can grow around and between soil minerals and organic particles and physically bind them together. The fungal filaments can stabilize soil structure as linear structures branch through the soil and wrap around soil particles, like, fishing nets [317].

Some microbes can absorb radiation and grow in disaster zones, such as Chernobyl and Fukushima [318]. High‐level radioactive waste sites contain a variety of microbial inhabitants, including bacteria and fungi. Surprisingly, many air‐sensitive bacteria (e.g., *Nocardia* and *Pseudomonas*) were isolated from the highly radioactive sediments at the Hanford facility, as well as species that were extremely resistant to infrared rays (e.g., *Deinococcus radiodurans*) [319]. Some radio‐resistant microbes can produce small organic molecules (extremolytes) to protect themselves and thrive under different types of radiation. Extremolytes (including scytonemin, mycosporine‐like amino acids, shinorine, porphyra‐334, palythine, biopterin, phlorotannin, etc.) can absorb a broad spectrum of radiation while protecting the organism's DNA from damage. Possible applications for these extremolytes include anticancer drugs, antioxidants, cell cycle blockers, and sunscreens [320].

### Physical to biological

Many physical exposures, such as light, temperature, and humidity, are necessary for organisms. Sustained high temperatures can destroy most bacteria or viruses; for example, the SARS‐CoV‐2 can be killed by 3‐min exposing to above 75°C, or lower temperatures for a longer time [321]. People usually treat milk and other foods with ultrahigh‐temperature processing to get rid of harmful microbes [322, 323]. Some organisms can capture energy from sunlight and use it to produce organic compounds, a process called photosynthesis [324]. For animals, sunlight can stimulate melanin production, linked to circadian rhythms [325, 326].

But exposure to UV from sunlight has adverse effects, as UV radiation is the most basic form of radiation. Radiation is energy in the form of electromagnetic waves (gamma rays, X‐rays, UV, radio waves, etc.), which causes oxidative damage to biomolecules, such as proteins, DNA, RNA, and enzymes. Excessive or intense exposure to radiation can induce a variety of mutagenic and cytotoxic DNA damage that can lead to different forms of cancer [320].

Taking advantage of the adverse effects of radiation on microorganisms, we can eliminate potential pathogens in environments. Germicidal ultraviolet (GUV) is widely used for potable water disinfection where its efficacy against a wide range of water‐borne pathogens is demonstrated [327]. It works primarily by causing damage to nucleic acids (DNA or RNA), universally present in pathogenic microbes. Its efficacy against protozoa, fungi, bacteria, and viruses is assured, with some variability in the dose required. Fungal spores are among the most challenging pathogens to inactivate, but GUV effectively reduces mold growth in air‐conditioning coils and drip pan surfaces. GUV is a valuable and necessary engineering intervention to reduce the transmission of COVID‐19 [327].

### Chemical to physical

Global warming is one of the most pressing environmental challenges faced by humanity. The main reason for the increase in atmospheric temperature is the excessive emission of greenhouse gases. Greenhouse gases are gaseous compounds that emit UV radiation in a specific thermal infrared range [328]. Greenhouse gases keep temperatures high in the lower atmosphere, allowing less heat to escape back into space. This, in turn, leads to the greenhouse effect and global warming. On the other hand, greenhouse gases are critical to maintaining a habitable temperature for the Earth, as the Earth's average surface temperature would be about −18°C if the atmosphere were completely free of greenhouse gases. Common greenhouse gases in the atmosphere include water vapor, chlorofluorocarbons (CFC), hydrofluorocarbons, CO_2_, CH_4_, nitrous oxide (N_2_O), and O_3_. However, the researchers point out that the four major greenhouse gases of global concern today are CO_2_, SO_2_, CH_4_, and N_2_O. While water vapor is arguably the most abundant naturally occurring greenhouse gas in the atmosphere, CO_2_ is the most emitted [328].

Some airborne pollutants (e.g., ozone and PM) can limit the level of UV radiation [329] and modify one's UV exposure. Tropospheric ozone and PM can absorb and scatter UVB, reducing the amount of radiation reaching the earth [330, 331]. Thus, once the ozone layer is destroyed, caused by the accumulation of ozone‐depleting chemicals, such as CFCs, organisms will be exposed to significantly more UV radiation [332]. In addition to the natural barrier of the ozone layer, certain chemicals are used to make UV‐blocking products that protect the skin from radiation. The FDA has approved 17 ingredients for use in sunscreens, including oxybenzone, titanium dioxide, and zinc oxide [333].

### Physical to chemical

Physical exposures, including temperature, humidity, wind speed, and solar radiation, have been widely considered in air pollution studies and have influenced the air chemical exposome [70]. For example, in a well‐ventilated urban space, the air pollution is relatively light, and the residence time of pollutants is short. Air pollution is high in high‐density and poorly ventilated areas, and residence time is extended. Certain wind speed and turbulent conditions may help remove air pollutants [334]. Both atmospheric mixing heights and chemical reaction rates can vary with temperature, thus disturbing PM composition. Pollutants' transportation can be altered by temperature changes and airflow patterns [335]. Relative humidity was identified as a regulator to explain the heterogeneity of pollution effect between cities. Air pollution has a more significant impact in drier countries [336]. UV irradiation can induce NO_*x*_ release into the circular system [337]. Solar radiation is also associated with generating secondary aerosols by accelerating photochemical reactions, which can distribute pollutants and exacerbate pollution [338].

In summary, biological, chemical, and physical exposures are interwoven to maintain the balance of the ecosystem and the homeostasis of life at the mechanistic level. Notably, the interactions among exposures are also being exploited for the benefit of society.

## OUTSTANDING CHALLENGES

**Figure 5:**
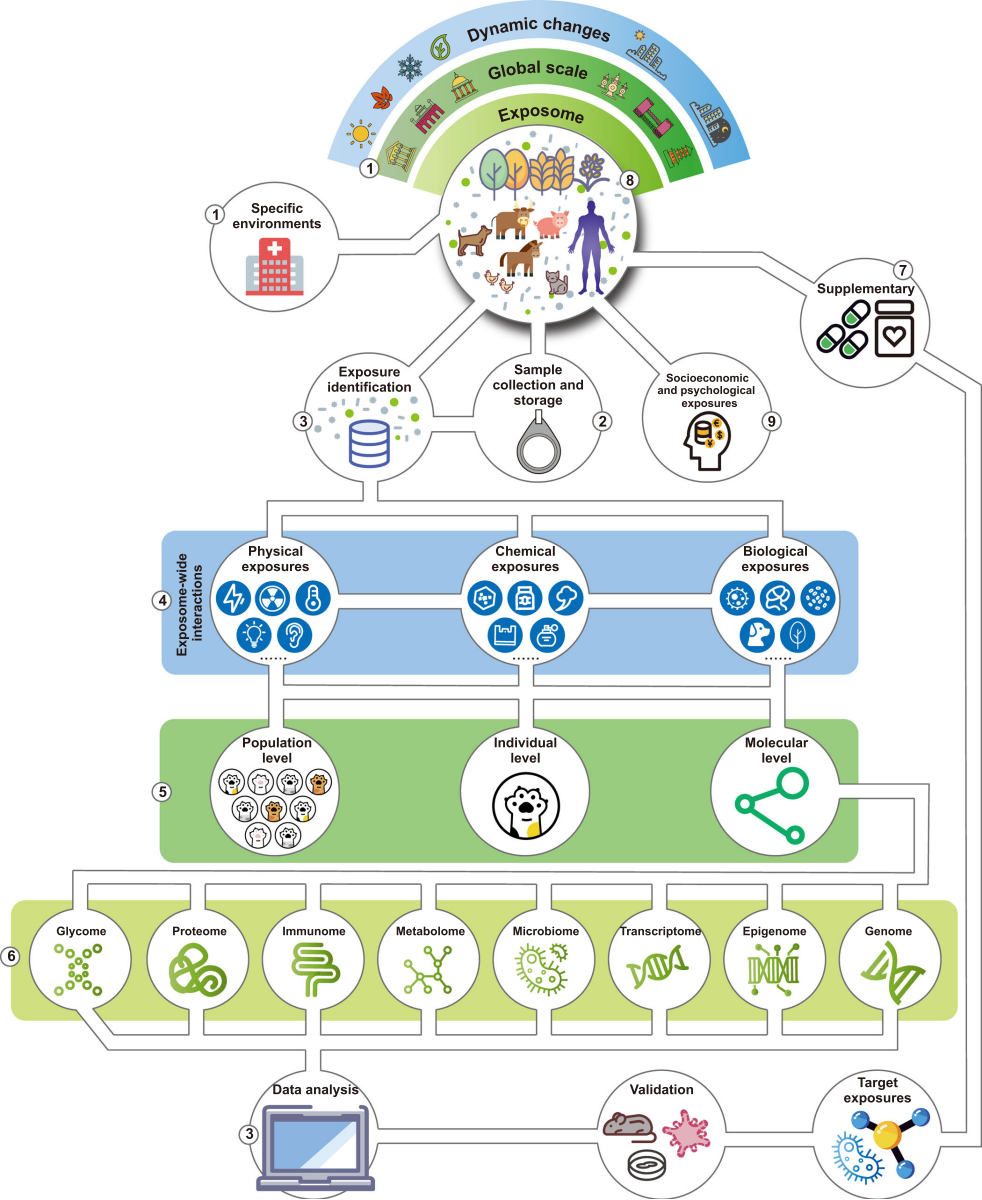
Outstanding challenges under the general exposome research framework. (1) The exposome is spatiotemporally dynamic. There is a distinct lack of exposome monitoring efforts at the systematic level. (2) Exposome sampling methods need further development. (3) Experimental and computational methods to monitor and identify exposures need further development. (4) Exposures can interact with each other; detailed interactions of exposures are understudied. (5) Effects of various environmental exposures at the population, individual, and molecular levels need to be evaluated systematically and quantitatively. (6) A multidimensional and interdisciplinary approach is needed to investigate how external environmental exposures are translated into internal exposures and responses. (7) A greater focus on beneficial exposures can provide valuable insights into designing new supplementary therapeutic strategies. (8) All organisms are equally impacted by the exposome. The impact of exposomes on other organisms, such as crop plants, stock animals, and pets, should be investigated. (9) The humanistic exposome is relatively understudied.

This review extensively summarized the biological and chemical environmental exposures in air, water, and soil environmental matrices and briefly covered the physical exposome. We also discussed how these exposures are potentially interconnected. While meticulously characterizing and quantifying the exposome components is the foundation of studying the exposome, we recognize that it is only the beginning. Below, we list some of the outstanding challenges we consider essential to address under a comprehensive exposome research framework to understand the impact of exposome on human, animal, plant, insect, and microbiome health (Figure 5):

1. There is a distinct lack of exposome monitoring efforts at a systematic level. Current studies usually focus on local or regional exposures. We need to take a more global approach to include more diverse and understudied regions. Detailed exposome monitoring of specific environments of interest, such as hospitals, daycare centers, factories, mining facilities, offices, and schools, is also lacking. The recent study on the surface microbiome of global urban subway and public transportation systems is an excellent example of a baseline understanding of the exposome on a worldwide scale [339]. Chemical and biological exposures are spatiotemporally dynamic and also constantly change at the molecular level. Biological species constantly mutate and evolve, acquiring resistant and toxic genes through horizontal gene transfer. We need to investigate the cellular mechanism of exposures incorporating evolutionary and inheritance frameworks [23].
2. Exposome sampling methods need further development. Devices to sample exposome in different environmental matrices are often stationary, cumbersome, large, and difficult to use. Portable or wearable devices to monitor exposomes in different environmental matrices would immensely empower this field. Several recent works have already used wearable devices to study the personal exposome [24, 39, 41, 42]. In the future, we need to develop more robust and intelligent devices. For example, we can design accessories for smartphones to sample exposures. Targeted real‐time detection of selected environmental exposures can be critical in needed situations. New technologies such as nanoflower can potentially be applied in the targeted real‐time detection devices to monitor key exposures of concerns [340].
3. Current methods of identifying biological and chemical exposures heavily depend on existing reference databases [341], and the understanding of physics directly limits our knowledge of physical exposures. Developing better experimental and computational methods to identify environmental exposures, especially those not included in the reference biological and chemical databases, is urgently needed. For example, machine learning algorithms can predict the chemicals based on MS data. Exposome research utilizes the physical, chemical, and biological approaches to provide a comprehensive view of the impacts of exposures. However, the data types and methods are mostly omics‐specific, making the integrated analysis difficult, inextensible, and irreproducible. We need to develop standardized and containerized data processing pipelines. We also need to develop tools to resolve the complex relationship between exposures and health status to identify the key exposures and the underlying mechanisms.
4. The understanding of detailed interactions of exposures is still lacking. All chemicals are potentially subjected to complex yet unknown reactions in the environment [186, 342, 343, 344, 345]. Computational methods have started to gain ground in understanding the interactions in environmental exposome to shed mechanistic insight at the atomistic/electronic level [346, 347]. In addition, some of the interactions are mediated by microbes in the environment, which can increase/decrease the toxicity of chemicals. Biologicals also have complex ecological interaction networks, partially revealed by the recent personal exposome study [39].
5. There is a pressing need to systematically and quantitatively evaluate the effects of environmental exposures at the population, individual, and molecular levels. Thanks to decades of efforts in environmental toxicology, infectious diseases, and, more recently, microbiome research, we now have a collection of established animal model systems. Given the 3Rs alternatives agenda, additional experimental model systems/technologies need to be further developed and incorporated into exposome studies, such as the organoids [348], three‐dimensional bioprinting [349], and organ‐on‐a‐chip [350]. However, our understanding of the impact of chemical exposures currently surpasses biological exposures, and scientists mainly focus on the acute consequences of biological exposures. The effects of long‐term exposures are investigated under the epidemiological framework but often lack mechanistic insights. The exposome framework can interface with and influence the field of molecular toxicology, which can help advance the exposome field by providing the needed mechanistic understanding of the exposome impacts on health [15]. As an example, it is commonly believed that in humans, exposure during early life can have a real and severe impact in the following decades, impacting the development of the immune system, which can lead to allergy/asthma/mental conditions, and so forth.
6. A multidimensional and interdisciplinary approach is needed to investigate how external environmental exposures are translated into internal exposures and responses, disturbing molecular interactions at the DNA, RNA, protein, and metabolite levels, leading to corresponding health outcomes. A recent study attempted to integrate the personal external and internal exposome and investigated the health outcome of individuals using multiomics [24]. The recent decade's translational achievements of microbiome research set an excellent example for this goal.
7. While the goal of exposome research is to identify all exposures contributing to the health trajectories of organisms, there is currently a stronger focus on the adverse exposures in the field of the environmental exposome. But exposures can also be helpful and necessary; for example, in addition to vaccines and medications, which can be viewed as a part of the broad exposome, there is now abundant evidence in the microbiome field that having certain species of microbes or metabolites in the gut would be beneficial for one's health. Exposures of other domains (i.e., chemical and physical) could also echo the findings in microbiome studies. It would be interesting to see how the idea of probiotics, prebiotics, and postbiotics can be expanded to cover all types of exposures relevant to health.
8. While current exposome research mostly focuses on humans, all organisms are equally impacted by the exposome [23]. Besides crop plants, stock animals, and pets directly related to human civilization, which warrants close investigations, exposome's impact beyond the human influence circle should not be overlooked. The effect of exposome is not limited to organisms [18, 351, 352]; for example, the integrity of materials, synthetic or natural, is heavily subjected to the influence of exposome, which would be an entirely different topic to cover in the realm of material science.
9. The humanistic exposome is relatively understudied. For socioeconomic and psychological exposures, better scope and quantification approaches are needed to facilitate the integrated study of environmental exposome with humanistic exposome.

As an example to illustrate the power of exposome research, a recently published study investigated the adverse impacts of endocrine‐disrupting chemicals (EDCs) from the population to the molecular level [353]. First, a mixture of EDCs (based on established knowledge databases) was identified based on their associations with adverse neurodevelopmental outcomes in a population‐scale data analysis using the Swedish environmental longitudinal, mother and child, asthma and allergy (SELMA) pregnancy cohort. Next, the identified EDCs were mixed to make the MIX N for subsequent use in the experimental systems. Although MIX N only represents a portion of all chemicals that humans are exposed to at present, it is still a significant advancement from the traditional single compound‐based approaches. At the molecular level, gene regulatory networks and cellular responses dysregulated by MIX N were characterized in human fetal neural progenitors and cortical brain organoids in vitro. The researchers then validated the key pathways affected by MIX N and their physiological impact in in vivo models, elucidating the molecular and functional impact of exposures. Finally, the impact of MIX N on prenatal development was extrapolated back to the population exposure data to construct a risk assessment scoring system. Taken together, this study integrated epidemiological and experimental data and established correlative and causal evidence for the health outcomes of exposure to a specific group of chemicals.

As more and more researchers from different fields are drawn into this exciting new frontier, we are optimistic that the original visions of the exposome research will be fulfilled, and we will come to a holistic understanding of health and diseases in humans and, indeed, any extant organisms.

## AUTHOR CONTRIBUTIONS

**Xin Wei**: Writing—original draft preparation, visualization, writing—reviewing and editing. **Zinuo Huang**: Writing—original draft preparation, visualization. **Liuyiqi Jiang**: Visualization, writing—reviewing and editing. **Yueer Li**: Software. **Xinyue Zhang**: Writing—reviewing and editing. **Yuxin Leng**: Writing—reviewing and editing. **Chao Jiang**: Conceptualization, writing—original draft preparation, writing—reviewing and editing.

## CONFLICT OF INTEREST

The authors declare no conflict of interest.

## Supporting information

Supplementary information.

## Data Availability

Biological and chemical exposure data used for the construction of the taxonomy trees are included in Supporting Information Tables S1 and S2. All supplementary materials (figures, tables, scripts, graphical abstract, slides, videos, etc.) may be found in the online DOI or iMeta Science http://www.imeta.science/.
